# Decreases in purchases of energy, sodium, sugar, and saturated fat 3 years after implementation of the Chilean food labeling and marketing law: An interrupted time series analysis

**DOI:** 10.1371/journal.pmed.1004463

**Published:** 2024-09-27

**Authors:** Lindsey Smith Taillie, Maxime Bercholz, Barry Popkin, Natalia Rebolledo, Marcela Reyes, Camila Corvalán

**Affiliations:** 1 University of North Carolina at Chapel Hill, Carolina Population Center and Gillings School of Global Public Health, Department of Nutrition, Chapel Hill, North Carolina, United States of America; 2 CIAPEC, Institute of Nutrition and Food Technology, University of Chile, Santiago, Chile

## Abstract

**Background:**

In 2016, Chile implemented a multiphase set of policies that mandated warning labels, restricted food marketing to children, and banned school sales of foods and beverages high in nutrients of concern (“high-in” foods). Chile’s law, particularly the warning label component, set the precedent for a rapid global proliferation of similar policies. While our initial evaluation showed policy-linked decreases in purchases of high-in, a longer-term evaluation is needed, particularly as later phases of Chile’s law included stricter nutrient thresholds and introduced a daytime ban on advertising of high-in foods for all audiences. The objective is to evaluate changes in purchases of energy, sugar, sodium, and saturated fat purchased after Phase 2 implementation of the Chilean policies.

**Methods and findings:**

This interrupted time series study used longitudinal data on monthly food and beverage purchases from 2,844 Chilean households (138,391 household-months) from July 1, 2013 until June 25, 2019. Nutrition facts panel data from food and beverage packages were linked at the product level and reviewed by nutritionists. Products were considered “high-in” if they contained added sugar, sodium, or saturated fat and exceeded nutrient or calorie thresholds. Using correlated random-effects models and an interrupted time series design, we estimated the nutrient content of food and beverage purchases associated with Phase 1 and Phase 2 compared to a counterfactual scenario based on trends during a 36-month pre-policy timeframe. Compared to the counterfactual, we observed significant decreases in high-in purchases of foods and beverages during Phase 2, including a relative 36.8% reduction in sugar (-30.4 calories/capita/day, 95% CI -34.5, -26.3), a 23.0% relative reduction in energy (-51.6 calories/capita/day, 95% CI -60.7, -42.6), a 21.9% relative reduction in sodium (-85.8 mg/capita/day, 95% CI -105.0, -66.7), and a 15.7% relative reduction in saturated fat (-6.4 calories/capita/day, 95% CI -8.4, -4.3), while purchases of not-high-in foods and drinks increased. Reductions in sugar and energy purchases were driven by beverage purchases, whereas reductions in sodium and saturated fat were driven by foods. Compared to the counterfactual, changes in both high-in purchases and not high-in purchases observed in Phase 2 tended to be larger than changes observed in Phase 1. The pattern of changes in purchases was similar for households of lower versus higher socioeconomic status. A limitation of this study is that some results were sensitive to the use of shorter pre-policy time frames.

**Conclusions:**

Compared to a counterfactual based on a 36-month pre-policy timeframe, Chilean policies on food labeling, marketing, and school food sales led to declines in nutrients of concern during Phase 2 of implementation, particularly from foods and drinks high in nutrients of concern. These declines were sustained or even increased over phases of policy implementation.

## Abstract 2

Lindsey Smith Taillie and team investigate if mandated front-of-package warning labels on foods and beverages lead to reduced purchases of nutrients of concern in Chili.

## Author summary

**Why was this study done?:**

- Chile implemented a multiphase set of policies to halt increases in obesity prevalence and noncommunicable disease risk.
- These policies mandated front-of-package warning labels on foods and beverages, restricted food marketing to children, and banned the sale of foods and beverages high in nutrients of concern (e.g., sugar, saturated fat) in schools.
- These policies were implemented in 3 phases, with increasingly strict nutritional thresholds in each phase and the introduction of a daytime marketing ban in Phase 2. We aimed to examine the pre-post association of these policies with food purchases during the Phase 2 implementation.

**What did the researchers do and find?:**

- Using longitudinal data on household food purchases, we estimated how purchases high in nutrients of concern were associated with the Phase 1 and Phase 2 implementations compared to a counterfactual scenario, or hypothetical food purchases if the policies had not been enacted, based on trends during a 36-month pre-policy timeframe.
- After the Phase 2 implementation, compared to the counterfactual, purchases of foods high in nutrients of concern decreased, ranging from approximately a 16% relative reduction for saturated fat purchases to a 37% relative reduction in sugar purchases, while purchases of foods not high in nutrients of concern increased. Compared to counterfactual, there were larger drops in purchases of nutrients of concern from high-in foods and beverages in Phase 2 compared to Phase 1; there were also larger increases in purchases from not high-in foods and beverages.
- There were few differences in post-policy food and beverage purchases by socioeconomic strata.
- Overall, there was a reduction in purchases of nutrients of concern during Phase 2 of implementation.

**What do these findings mean?:**

- The Phase 2 implementation led to declines in purchases of nutrients of concerns, especially from high-in foods and drinks.
- Purchasing changes of high-in and not-high in foods were more pronounced in Phase 2 than in Phase 1, suggesting that post-policy purchasing changes were maintained or even strengthened over time.
- Reductions in nutrients of concern were similar across socioeconomic strata, suggesting that this policy did not disproportionately advantage or disadvantage any group.
- The main limitations of this study are that the data included are only a portion of what Chilean households purchase, and total dietary intake data are needed to fully evaluate the policies.

## Introduction

In the last decade, many countries across the globe have taken action to halt increases in obesity prevalence and noncommunicable disease risk by implementing policies designed to reduce consumption of unhealthy packaged foods and sugar-sweetened beverages [1]. In the years since Chile first began implementing its landmark policies on front-of-package labeling, restricted marketing, and ban of school sales of food and drinks high in calories, added sugar, sodium, and saturated fat (“high-in” foods), many countries around the globe have followed suit. Chile’s policy requiring mandatory warning labels on the front of “high-in” foods has seen particularly rapid momentum, with the majority of countries in South and North America now having implemented or in the process of implementing similar policies. Even in the United States, where policy progress has been slower, the 2022 National White House Conference on Hunger and Health recommended clearer front-of-package labels as an important strategy for improving public health nutrition, and the US Food and Drug Administration is now researching design options for a simple, interpretive front-of-package food label, including a version of warning labels.

The Chilean regulations were implemented in 3 phases (2016, 2018, and 2019). Initial evaluations of the Chilean policy after the first phase found that it was associated with changes in the food environment, including reductions of nutrients of concern in the food supply (e.g., energy, sugar, sodium, and saturated fat) [2] and reductions in children’s exposure to unhealthy food advertising and marketing [3–7], improvements in mothers’ understanding of unhealthy foods and use of the warning labels [8], reductions in purchases of foods and drinks carrying the warning label [9–11], improvements in the nutritional quality of foods available in schools [12], and improvement in children’s dietary intake [13].

However, much less is known about policy-linked changes in dietary behavior over a longer time period. Evaluations of beverage taxes have shown sustained decreases in purchases 2 years after policy implementation [14–16], but to our knowledge, there have been no longer-term evaluations of a multicomponent nonfiscal policy such as Chile’s. One concern is that the impact of certain parts of the law like the warning labels might wear off over time as consumers became accustomed to seeing the labels on packages, reducing the influence on food purchases. On the other hand, the second phase of Chile’s law, implemented in July 2018, included important changes to the regulations that could have increased the law’s impact on food purchases. Nutrient thresholds (particularly for foods) became stricter, meaning that more foods and beverages were affected by the regulations. In addition, the marketing restriction expanded to include the first-ever daytime ban on television advertising of ***all*** “high-in” products to all audiences, not just those using child-directed appeals or appearing on children’s programs. While a recently published study found a 63% reduction in high-in food advertisements on television during Phase 2 [17], changes in food purchasing during this period are currently unclear.

Other questions relate to how the policy influenced specific product types. For example, it is unclear which food or beverage groups were most affected by the law, which is important for understanding nutrient changes and, ultimately, dietary patterns. In addition, it is unclear the extent to which the policy shifted purchases from products with multiple nutrients of concern (and thus likely to carry multiple warning labels) to products with one nutrient of concern (one warning label) or no nutrients of concern (no warning labels). These data are relevant to inform front-of-package labeling policies in other countries, who are grappling with the ideal number of warning labels to put on products in order to nudge consumers to healthier choices.

Finally, it is also important to understand whether the policies differentially influenced food purchases for households with low socioeconomic status (SES). A recent review of interpretive front-of-package labels found that while such labels are better understood than back-of-package nutrition information, relative to high-SES individuals, low-SES individuals are less likely to understand and use front-of-package labels and also less likely to shift purchasing intentions as a result of label exposure [18]. In addition, food prices are a major driver of food choices in low-SES households [19], potentially making them less likely to respond to nonfiscal policies like labeling and marketing policies. Understanding policy-linked purchasing changes among low-SES households is critical for ensuring future policies promote healthier diets for the entire population.

The objective of this study is to examine the pre-post association of the Chilean Law of Food Labeling and Advertising with purchases of nutrients of concern during Phase 2 of implementation of the law. A secondary objective is to explore whether changes in purchases over time differed by household SES and by number of nutrients of concern.

## Methods

This study was exempt from review by the University of North Carolina, Chapel Hill Institutional Review Board (IRB) as it used secondary, deidentified data. The study was approved by the University of Chile IRB.

### Study design and participants

We used longitudinal data on household food purchases from Kantar WorldPanel Chile (Kantar) from July 1, 2013 to June 25, 2019. Data were aggregated at the household-monthly level.

Households were excluded if they were missing demographic information for a given year. We also excluded all purchases in bulk (no quantity), purchases with a price or quantity of zero, baby food and formula, Kantar categories and subcategories that were introduced or discontinued during the study period as determined by changes in the number of purchases in these categories and subcategories over time (condensed milk, snacks, cereal bars, and minced meat), and household-month observations with total energy of zero across all food and beverage purchases. We also excluded household-month observations from new panelists before their first full month in the panel. We did so because Kantar began integrating new panelists around the last week of each month in February 2017, resulting in systematic measurement error in their first monthly totals. S1 Table shows each exclusion criteria and the number of household-month observations dropped. The final analytical sample included 2,844 unique households (median follow-up, 64 months; 138,391 household-month observations) from cities (population >20,000) across 13 of Chile’s 16 regions. The Kantar sample is weighted to be representative of these regions’ urban populations in terms of key demographics. Compared to the urban population nationwide (S2 Table), Kantar households are slightly more educated, bigger by just over one person on average, younger (main shopper’s age and head of household’s age), and similarly distributed geographically.

Data on household purchases included the volume (ml) or weight (g), barcode, date, price, retail outlet, brand, and package size. Data were then linked at the product level to nutrition data, which were collected and updated annually from 2015 to 2019. S3 Table reports the food and beverage categories included and their aggregate expenditure shares.

Data also included household characteristics, including size, composition (age and gender of each member), assets (number of rooms, bathrooms, and cars), geographical region, age of main shopper, head of household (primary earner) current educational attainment, and SES. Regarding the SES measure, households were categorized by Kantar into 4 categories using the 2007 methodology of the market research association *Asociacion de Investigadores de Mercado y Opinion Publica de Chile* (AIM), combining the lowest 2 groups into 1. Briefly, households were scored on whether they possessed certain goods (e.g., refrigerator, washing machine, DVD player) and paid for certain services (namely, internet and full-time domestic worker), as well as on the household head’s education level. The cutoff points for AIM’s 5 SES categories correspond to the 10th, 45th, 70th, and 90th percentiles of scores in a large sample of households in the Gran Santiago area conducted in 2007. One limitation of this methodology is that goods and services that could be used to reliably differentiate between SES groups in 2007 were not necessarily as relevant by the end of the study period. Therefore, we consider the SES indicator an imperfect SES measure.

We further linked the data to the quarterly regional unemployment rate [20] (downloaded from Chile’s statistics office’s website in September 2022) and public holiday data from the Python package “holidays” (version 0.19, with minor corrections).

For main analyses, the pre-policy period was defined as July 1, 2013 to June 30, 2016, Phase 1 was defined as July 1, 2016 to June 30, 2018, and Phase 2 was defined as July 1, 2018 to June 25, 2019. Because Chile experienced national civil unrest in October 2019 followed by the Coronavirus Disease 2019 (COVID-19) pandemic in February to March of 2020, we were unable to include Phase 3 in this study (beginning July 1, 2019) due to the food supply disruptions.

### The Chilean regulation

Chile’s law requires that packaged foods and beverages containing added sugar, added sodium, or added saturated and exceeding thresholds for these nutrients or overall calories carry front-of-package warning labels. The labels consist of black octagon(s) with the text that it is high in sugar, sodium, saturated fat, and/or calories. The products are also subject to marketing restrictions (disallowed to use child-directed marketing techniques or air on platforms targeting children). The products are also banned from sales or promotion in schools and nurseries. The policy was implemented in phases with increasingly strict nutrient thresholds implemented in July 2016 (Phase 1); July 2018 (Phase 2); and July 2019 (Phase 3) (S4 Table). The main difference between the Phase 1 and Phase 2 regulations was that for foods, the thresholds became much stricter for sodium (800 mg/100 g to 500 mg/100 g) and total sugars (22.5 g to 15 g/100 g), with smaller reductions for calories (350 calories to 300 calories per 100 g) and saturated fat (6 g/100 g to 5 g/100 g). For beverages, changes in the thresholds were smaller and affected only calories (from 100 calories/100 ml to 80 calories/100 ml) and sugar (from 6 g/100 ml to 5 g/100 ml). The other key difference between Phase 1 and Phase 2 is Phase 2 expanded the marketing restrictions to include a ban on advertising of products with the warning labels on daytime television from 6 AM to 10 PM, regardless of audience (in other words, it was no longer just limited to children’s programming).

### Nutritional data and linkage procedure

In order to estimate the nutrient content of food purchases in each policy period, the purchases data are linked to updated nutrition data on the food supply. As previously described, household purchases were linked at the product level to nutrition facts panel (NFP) data from food packages collected by the Institute of Nutrition and Food Technology (INTA), University of Chile via photographs taken in supermarkets in the first quarter of each year starting in 2015, as well as other data sources when INTA data were not available. As in previous studies, data were matched on barcode, and product characteristics (e.g., brand, product description) if no barcode match was found [9,21].

A visual depiction of linkages is available in S1 Fig, with more detail provided in S5 Table. In brief, for the pre-policy period, purchases were primarily linked to NFP data collected in the first quarters of 2015 and 2016; for Phase 1, purchases were primarily linked to NFP data collected in the first quarters of 2017 and 2018; and for Phase 2, purchases were primarily linked to NFP data collected in the first quarter of 2019. To link unmatched products, we extended the search to INTA data from previous years, starting with 1 year prior, then 2 years prior, etc., down to 2015. Any remaining unmatched products were linked to Mintel’s New Products Database for the Americas (mostly Chile), and other sources for a very small number of products.

As with any linkage procedure, this linkage assumes that the nutrient content of products was constant within each linked period (pre-policy, parts 1 and 2 of Phase 1, and Phase 2), rather than what was more likely, that changes in nutrient content occurred over time as products were gradually reformulated, new products were introduced, and old products were removed. In particular, for purchases linked to the preferred NFP data year (e.g., 2019 for Phase 2 purchases), reformulations that occurred during the linked period appear as if they occurred either at the start of the period (if reformulated *before* the NFP data were collected) or at the start of the *following* period (if reformulated *after* the NFP data were collected). For example, a product purchased in Q3 of 2016 and linked to NFP data collected in Q1 of 2017 is assumed to have the same nutrient content as it did in Q1 of 2017, even though it could have been reformulated *after* the purchase (thereby understating nutrients of concern, if they were reduced by reformulation). Conversely, a product purchased in Q2 of 2017 and linked to the same NFP data is assumed to have the same nutrient content as it did in Q1 of 2017, even though it could have been reformulated *before* the purchase (thereby overstating nutrients of concern, if they were reduced by reformulation).

#### Classification of products as “high in” or not “high in”

All purchases were classified as to whether they were “high in” or “not high in” nutrients of concern according to the final thresholds established in the final phase (Phase 3) of the Chilean regulation. We decided to use the Phase 3 thresholds and apply them across all time periods, instead of applying the corresponding nutrition thresholds in each phase, for multiple reasons: (1) this is the set of products that the final Chilean law ultimately deemed unhealthy and thus is the set of products we wish to track over time; (2) to retain consistency in definition across the entire time period, so that our estimation represents changes in *purchases* rather than changes in *thresholds*. It was not possible to conduct the analysis according to whether products actually contained labels because, by definition, no products carried labels in the pre-policy period (at least until closer to the start of Phase 1). Thus, the “high-in” designation reflects the products that *would* be subject to carry warning labels and be subject to marketing restrictions and school sales bans according to the final Chilean regulation.

Specifically, nutritionists reviewed each product for nutritional accuracy and, for consistency across all policy periods, applied the Phase 3 thresholds to categorize each product as "high-in” if it contained added sugar, sodium, or saturated fat and exceeded nutrient or calorie thresholds in Phase 3 and thus was subject to the labeling, marketing, and school regulations [22]. Foods and beverages were categorized as not high-in if they contained no nutrients of concern or they did not exceed the thresholds for these nutrients.

Nutritionists also categorized foods into nutritionally and behaviorally relevant groups (S3 Table). Some groups were excluded from analysis because they were not affected by the regulation (e.g., plain teas, sweeteners, vegetable oils, formulas, and supplements).

### Outcomes

All outcomes were standardized per capita per day by taking sums at the household-month level and dividing by the number of days in the month and number of household members. As in our previous evaluation, the main outcome was total energy (calories), which was selected as the primary outcome due to its relevance for obesity prevention, one of the main goals of the law. We also examined total sugar (calories), saturated fat (calories), and sodium (mg). For all nutrients, we conducted analyses of overall purchases and for high-in and not high-in purchases. Consistent with our first-phase evaluation [9], we also examined food (all 4 outcomes) and beverage (energy and sugar) purchases separately and by subgroup (restricting our attention to subgroups accounting for at least 5% of aggregate food or beverage expenditure and with a relatively high proportion of high-in products).

Lastly, we wanted to understand how purchases of products changed by whether they were high in 0, 1, or 2 or more nutrients of concern (and thus likely to carry 0, 1, 2, or more warning labels). To do this, we also aggregated purchases of products with 0, 1, or 2 high-in nutrients at the household-month level and then calculated daily per capita values.

### Statistical analysis

Statistical code for all analysis is available on Open Science Framework: https://osf.io/fcp9r/.

We used an interrupted time series design with immediate intercept and slope changes and an exponential conditional mean to estimate the changes in the nutritional content of food and beverage purchases associated with the first and second phases of the law, i.e., we assumed that each phase was associated with immediate relative changes (represented by the intercept) and that these changes may have increased or decreased over time (represented by the slope). These assumptions reflect both the nature of the intervention and the way we linked the purchase and NFP data. First, we assumed relative changes in purchases because absolute changes are likely to differ across households with varying levels of nutrients of concern. Second, because of how we linked the purchase and NFP data, reformulations appear as if they all occurred in July 2016 (start of Phase 1), July 2017, and July 2018 (start of Phase 2), hence the assumption of immediate changes in nutrients of concern relating to reformulation of nutrients in products. Third, we allowed these changes to increase or decrease over time to reflect reformulations mid-Phase 1 (July 2017) and gradual behavioral change on the part of consumers.

We used correlated random-effects exponential mean (Poisson) models together with the quasi-likelihood Poisson estimator with standard errors clustered at the household level to estimate the parameters of interest (model and equations can be found in S1 Appendix). The impact model took the form of a continuous variable for time (at monthly intervals), pre-post indicator variables for the first and second phases, and interactions with time. Although the study period is relatively short, we considered a quadratic time component, but the results were highly implausible (not reported). We controlled for seasonality by including indicator variables for each month of the year (reference: January), number of household members by age and sex (children aged 0 to 1 year, 2 to 5 years, and 6 to 13 years, females aged 14 to 18 years and 19 years or over, and males aged 14 to 18 years and 19 years or over), SES (ABC1, C2, C3, and DE; reference: ABC1), the head of household’s education level (less than high school, high school, and more than high school; reference: less than high school), the unemployment rate at the region and quarter level, the number of public holidays at the region and month level, and a pre-post indicator variable for the SSB tax implemented on October 1, 2014. The correlated random effects were implemented using Mundlak’s device, i.e., by adding the within means of all time-varying variables.

We then used the regression results to derive estimates of the average percentage and absolute changes associated with each phase of the law, calculated as differences in predicted values between the actual scenario and the counterfactual in which the law had not passed. We also estimated monthly means in the actual and counterfactual scenarios to depict these differences over time. We weighted these estimates using Kantar’s annual projection weights.

Separately, we created an indicator variable for low SES by combining the lowest 2 levels and interacted it with each variable of the impact model to see if SES moderated the law’s impact. For these analyses, we focused on energy as our main outcome of interest. For these models, we dropped the head of household’s education level from the model because it is a component of SES, so keeping it would have meant restricting any moderation effect to the other components of SES.

To understand whether there were bigger changes for products that were high in more nutrients of concern, we also examined the primary outcome by the number of nutrients of concern in excess. Finally, to understand if different food groups changed more or less after the law’s implementation, we conducted analyses using the same model as in the main analyses to examine nutrient outcomes for food and beverage subgroups.

### Sensitivity analyses

We conducted a series of sensitivities analyses, focusing on energy outcomes. First, we explored differences in estimates when allowing for a label rollout period between April and June 2016 (i.e., assuming that some companies may have placed warning labels on products before the official implementation date) [23,24]. We did so by adding a temporary slope change (but no intercept change) during the rollout period, assuming that it would not have occurred absent the policy. Second, we reduced the length of the baseline period from 36 months (which balances the pre and post periods) to 30, 24, and 18 months by dropping observations before January 2014, July 2014, and January 2015, respectively. These analyses allow us to understand how the use of different baseline periods may affect results (either through changes to trends in purchasing behaviors, or changes to assumptions around reformulation, or both).

Since there were differences in the distribution of current educational attainment levels in the Kantar WorldPanel sample over time compared to estimates of educational attainment for urban households from the nationally representative CASEN (Encuesta de Caracterización Socioeconómica) (S6 Table), we also conducted several sensitivity analyses on education. First, we estimated energy outcomes in models without adjusting for current household educational attainment. In a subsequent model, we included *initial* household head educational attainment in the model (e.g., we adjusted for the first observed value of educational attainment), since *initial* education was more consistent to CASEN estimates over time than was *current* education.

Lastly, although the Poisson model is well suited for nonnegative skewed dependent variables, we compared our food and beverage group results to estimates obtained from a 2-part model consisting of a correlated random-effects logit model in the first part (with the same explanatory variables) and the correlated random-effects Poisson model for the nonzero outcomes in the second part. All but the baseline period and 2-part model sensitivity analyses were added in response to reviewers’ comments.

All analyses were conducted in Stata 17 (College Station, Texas, USA).

#### Role of the funding source

Study funders had no role in any aspect of the study, from design to interpretation to publication. Furthermore, none of the coauthors have any conflict of interest.

## Results

Descriptive characteristics of the sample can be found in Table 1. Household size increased over time; although household SES remained consistent over time, the household head’s current educational attainment level increased.

**Table 1:** Weighted sociodemographic characteristics of the Kantar WorldPanel sample by year.

	2013	2014	2015	2016	2017	2018	2019
Household size	4.2	4.4	4.2	4.2	4.3	4.4	4.5
	(1.6)	(1.7)	(1.6)	(1.7)	(1.7)	(1.7)	(1.7)
Main shopper’s age	47.9	48.3	48.2	48.6	49.3	49.4	49.6
	(14.5)	(14.7)	(15.0)	(14.9)	(15.2)	(15.5)	(15.5)
Number of cars	0.5	0.6	0.7	0.7	0.7	0.7	0.7
	(0.7)	(0.7)	(0.8)	(0.7)	(0.7)	(0.7)	(0.8)
Number bathrooms	1.3	1.3	1.3	1.3	1.3	1.3	1.3
	(0.6)	(0.6)	(0.6)	(0.6)	(0.6)	(0.6)	(0.7)
Number bedrooms	2.9	3.0	3.1	3.0	3.0	3.0	3.1
	(1.0)	(2.4)	(1.5)	(1.0)	(1.0)	(1.0)	(1.0)
Household head’s current education							
Lower than high school	42%	40%	37%	32%	31%	28%	27%
High school	37%	38%	40%	42%	42%	45%	44%
Higher than high school	21%	22%	23%	25%	27%	28%	29%
Socioeconomic status (SES)							
ABC1 (highest)	8%	8%	8%	8%	8%	8%	7%
C2	17%	17%	17%	16%	16%	17%	16%
C3	27%	27%	27%	27%	27%	27%	28%
DE (lowest)	49%	49%	49%	49%	49%	48%	49%
Region							
North	13%	13%	12%	13%	13%	13%	13%
Center	64%	63%	63%	63%	63%	62%	62%
South	24%	24%	24%	24%	24%	25%	25%
Number of household-month obs.	11,636	23,455	23,292	23,385	22,762	22,684	11,177

Notes: The sample starts in July 2013 and ends in June 2019. The definition of SES is based on Chile’s Association for Market Research and Public Opinion (*Asociacion de Investigadores de Mercado y Opinion Publica*), which is a measure of SES based on a list of goods and services consumed by the household and the education level of the head of household.

Prior to the law, approximately 44% of foods were eligible to carry 2 or more warning labels, 25% were eligible to carry 1 warning label, and 31% would have had 0 warning labels (S7 Table). For beverages, only 3% were eligible to carry 2 or more warning labels, 39% were eligible to carry 1 warning label, and 58% would have had 0 warning labels.

### Unadjusted results

Unadjusted results on household purchases can be found in S8 and S9 Tables. The pattern of results showed a decline in nutrients of concern purchased across the study time period as well as declines in the share of nutrients from purchases of high-in products, ranging from -5.6 percentage points for saturated fat to -16.5 percentage points for sugar.

### Adjusted results

**Fig 1:**
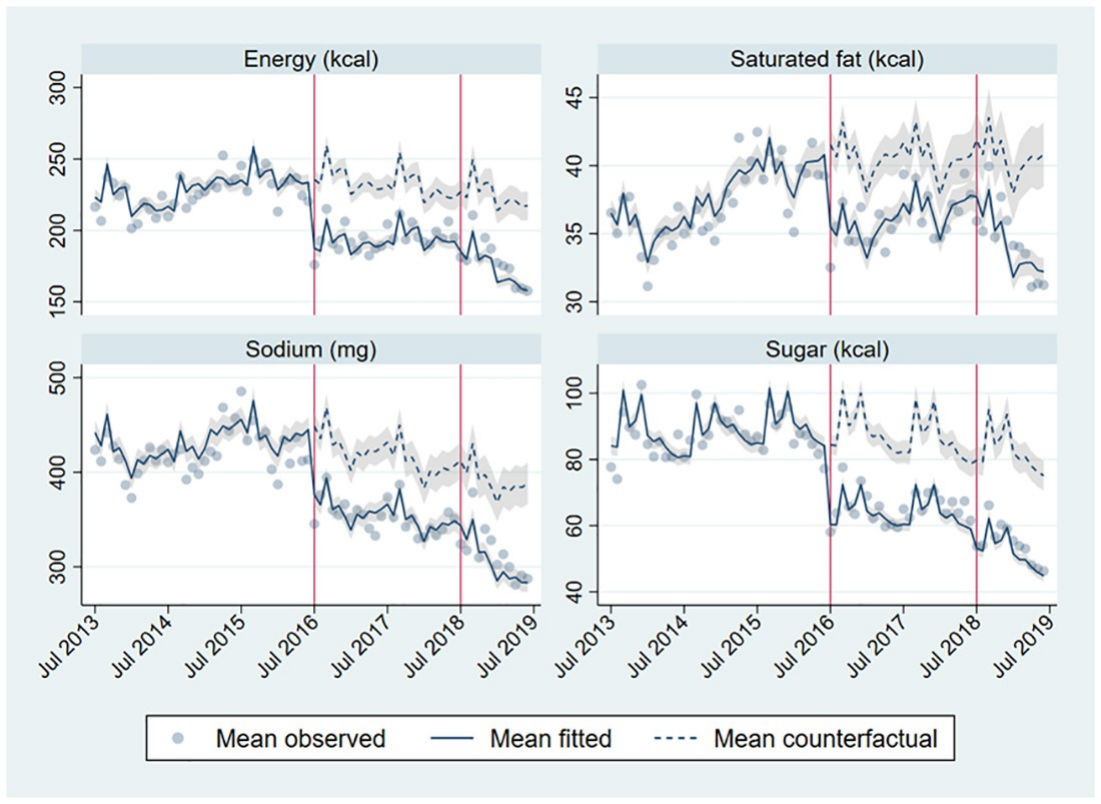
Mean observed, fitted, and counterfactual energy, saturated fat, sodium, and sugar per capita per day from high-in food and beverage purchases. Adjusted for seasonality (month dummies), household composition (number of household members by age and sex group), SES (4 categories), head of household education level (less than high school, high school, more than high school), region-quarter unemployment rate, number of public holidays in the month, October 2014 beverage tax changes (pre-post dummy), and unobserved time-invariant household characteristics. *N* = 138,367 household-month obs. (2,842 households).

**Fig 2:**
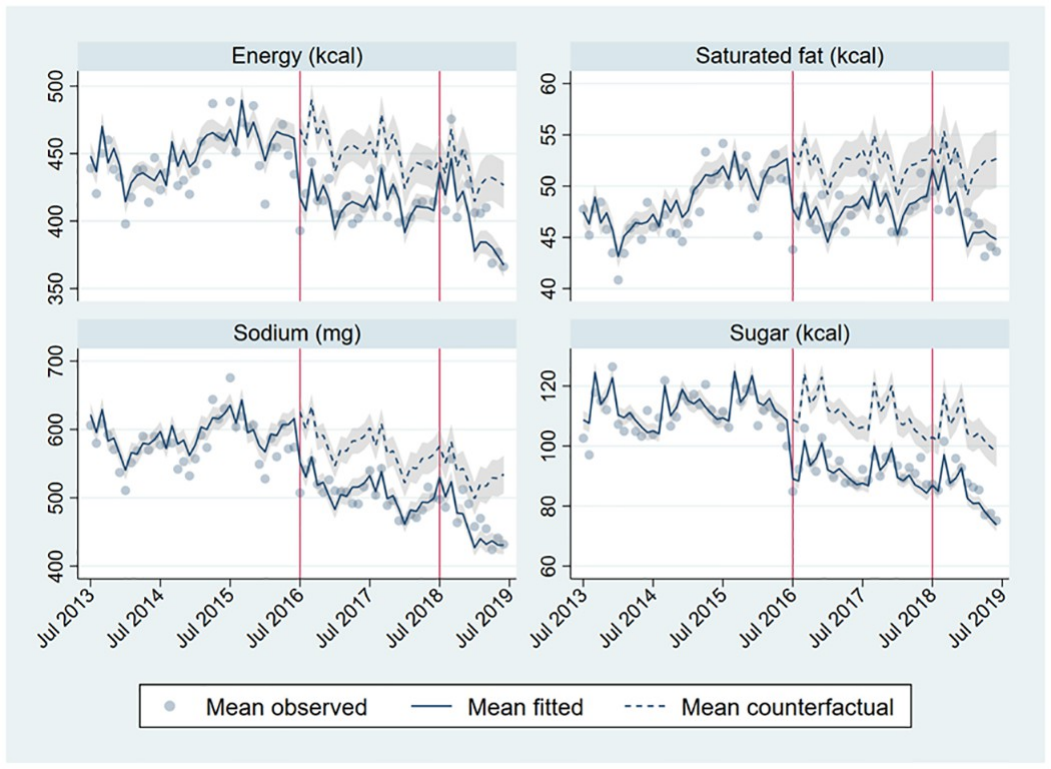
Mean observed, fitted, and counterfactual energy, saturated fat, sodium, and sugar per capita per day from total food and beverage purchases. Adjusted for seasonality (month dummies), household composition (number of household members by age and sex group), SES (4 categories), head of household education level (less than high school, high school, more than high school), region-quarter unemployment rate, number of public holidays in the month, October 2014 beverage tax changes (pre-post dummy), and unobserved time-invariant household characteristics. *N* = 138,367 household-month obs. (2,842 households).

Comparing weighted estimates of purchases as observed to the counterfactual scenario in Phase 1 and Phase 2, we observe significant decreases in purchases of high-in foods and beverages during Phase 2 (Figs 1 and 2), including a 36.8% relative reduction in sugar (-30.4 calories, 95% CI -34.5, -26.3), a 23.0% relative reduction in energy (-51.6 calories, 95% CI -60.7, -42.6), a 21.9% relative reduction in sodium (-85.8 mg, 95% CI -105.0, -66.7), and lastly, a 15.7% relative reduction in saturated fat (-6.4 calories, 95% CI -8.4, -4.3).

These decreases were partially compensated by increases in not high-in purchases (Table 2), but there remained a significant decrease in total nutrients purchased during Phase 2, resulting in relative reductions of 20.2% in sugar (95% CI -23.8, -16.7), 13.8% in sodium (95% CI -17.6, -9.9), -9.6% in saturated fat (95% CI -13.8, -5.3), and 8.3% in energy (95% CI -11.6, -5.0) from total food and beverage purchases.

**Table 2:** Estimated average absolute and relative changes from the no-policy counterfactual by phase and high-in status.

	Energy (kcal/capita/day)		Saturated fat (kcal/capita/day)		Sodium (mg/capita/day)		Sugars (kcal/capita/day)	
	Abs. diff.	% diff.	Abs. diff.	% diff.	Abs. diff.	% diff.	Abs. diff.	% diff.
**Total**								
Phase 1								
High-in	**-40.0**	**-17.1%**	**-4.4**	**-10.9%**	**-64.0**	**-15.2%**	**-23.4**	**-26.7%**
	(-45.1,-34.9)	(-18.9,-15.3)	(-5.5, -3.3)	(-13.3, -8.5)	(-75.5,-52.5)	(-17.6,-12.9)	(-25.9,-20.9)	(-28.8,-24.6)
Not high-in	**-0.1**	**0.0%**	**-0.1**	**-0.6%**	**-3.4**	**-2.2%**	**3.3**	**13.8%**
	(-5.2, 5.0)	(-2.4, 2.3)	(-0.6, 0.4)	(-4.9, 3.8)	(-10.2, 3.4)	(-6.5, 2.1)	(2.5, 4.1)	(10.0, 17.6)
Total	**-40.0**	**-8.8%**	**-4.5**	**-8.6%**	**-66.7**	**-11.6%**	**-19.5**	**-17.6%**
	(-48.5,-31.5)	(-10.5, -7.1)	(-5.8, -3.2)	(-10.9, -6.3)	(-80.4,-53.0)	(-13.7, -9.5)	(-22.3,-16.8)	(-19.6,-15.6)
Phase 2								
High-in	**-51.6**	**-23.0%**	**-6.4**	**-15.7%**	**-85.8**	**-21.9%**	**-30.4**	**-36.8%**
	(-60.7,-42.6)	(-26.2,-19.8)	(-8.4, -4.3)	(-19.9,-11.4)	(-105.0,-66.7)	(-25.8,-18.0)	(-34.5,-26.3)	(-40.1,-33.6)
Not high-in	**15.1**	**7.1%**	**1.4**	**11.8%**	**10.7**	**7.3%**	**8.3**	**35.4%**
	(5.6, 24.6)	(2.3, 11.9)	(0.4, 2.3)	(2.9, 20.7)	(-0.4, 21.8)	(-0.9, 15.4)	(6.8, 9.8)	(27.0, 43.8)
Total	**-36.2**	**-8.3%**	**-5.0**	**-9.6%**	**-73.5**	**-13.8%**	**-21.3**	**-20.2%**
	(-51.8, -20.6)	(-11.6, -5.0)	(-7.5, -2.6)	(-13.8, -5.3)	(-97.2, -49.9)	(-17.6, -9.9)	(-25.9,-16.7)	(-23.8,-16.7)
**Foods**								
Phase 1								
High-in	**-17.5**	**-10.0%**	**-3.8**	**-9.6%**	**-55.4**	**-13.8%**	**-2.9**	**-8.6%**
	(-21.5,-13.6)	(-12.1, -8.0)	(-4.8, -2.7)	(-12.0, -7.1)	(-66.7,-44.1)	(-16.3,-11.4)	(-3.8, -1.9)	(-11.2, -6.0)
Not high-in	**-2.0**	**-1.1%**	**0.5**	**12.3%**	**-3.3**	**-2.9%**	**0.7**	**10.7%**
	(-6.4, 2.5)	(-3.6, 1.4)	(0.2, 0.7)	(4.6, 20.0)	(-9.6, 2.9)	(-8.4, 2.5)	(0.4, 1.0)	(5.9, 15.5)
Total	**-19.6**	**-5.5%**	**-3.3**	**-7.6%**	**-58.2**	**-11.3%**	**-1.9**	**-4.8%**
	(-26.5,-12.7)	(-7.4, -3.7)	(-4.4, -2.2)	(-10.0, -5.2)	(-71.1,-45.3)	(-13.6, -9.1)	(-2.9, -0.9)	(-7.3, -2.3)
Phase 2								
High-in	**-22.6**	**-13.4%**	**-5.4**	**-13.6%**	**-73.7**	**-19.8%**	**-4.3**	**-14.2%**
	(-29.9,-15.4)	(-17.2, -9.7)	(-7.4, -3.4)	(-18.1, -9.2)	(-92.5,-55.0)	(-23.9,-15.7)	(-5.9, -2.7)	(-18.7, -9.7)
Not high-in	**10.0**	**5.9%**	**1.7**	**46.6%**	**8.3**	**7.8%**	**2.6**	**34.0%**
	(1.7, 18.3)	(0.7, 11.0)	(1.3, 2.2)	(29.2, 63.9)	(-1.7, 18.2)	(-2.5, 18.1)	(1.9, 3.2)	(23.2, 44.8)
Total	**-12.8**	**-3.8%**	**-3.5**	**-8.2%**	**-64.2**	**-13.5%**	**-1.1**	**-2.9%**
	(-25.7, 0.1)	(-7.5, -0.1)	(-5.6, -1.4)	(-12.6, -3.7)	(-86.4,-42.0)	(-17.6, -9.5)	(-2.8, 0.7)	(-7.6, 1.8)
**Beverages**								
Phase 1								
High-in	**-23.7**	**-39.5%**	n/a	n/a	n/a	n/a	**-20.9**	**-38.1%**
	(-26.0,-21.4)	(-42.0,-37.1)	n/a	n/a	n/a	n/a	(-23.1,-18.8)	(-40.6,-35.6)
Not high-in	**1.8**	**4.1%**	n/a	n/a	n/a	n/a	**2.4**	**13.6%**
	(0.1, 3.5)	(0.0, 8.1)	n/a	n/a	n/a	n/a	(1.7, 3.1)	(9.3, 18.0)
Total	**-20.9**	**-20.3%**	n/a	n/a	n/a	n/a	**-17.8**	**-24.7%**
	(-23.9,-17.9)	(-22.6,-18.0)	n/a	n/a	n/a	n/a	(-20.1,-15.6)	(-27.0,-22.4)
Phase 2								
High-in	**-30.6**	**-53.6%**	n/a	n/a	n/a	n/a	**-26.6**	**-50.8%**
	(-34.4,-26.8)	(-57.1,-50.2)	n/a	n/a	n/a	n/a	(-30.1,-23.1)	(-54.4,-47.2)
Not high-in	**5.0**	**11.9%**	n/a	n/a	n/a	n/a	**5.1**	**31.3%**
	(2.0, 8.1)	(4.0, 19.9)	n/a	n/a	n/a	n/a	(3.9, 6.3)	(22.0, 40.5)
Total	**-24.1**	**-24.5%**	n/a	n/a	n/a	n/a	**-20.4**	**-30.0%**
	(-29.2,-19.1)	(-28.4,-20.5)	n/a	n/a	n/a	n/a	(-24.1,-16.8)	(-33.9,-26.1)

Notes: 95% confidence intervals in parentheses. The absolute difference estimates are averaged over household-month observations in the relevant post-policy periods. The relative difference estimates are averages of month-specific coefficients. This is because the model yields the same predicted relative changes across all households in a given month but varies over time (due to the slope change). Adjusted for seasonality (month dummies), household composition (number of household members by age and sex group), SES (4 categories), head of household education level (less than high school, high school, more than high school), region-quarter unemployment rate, number of public holidays in the month, October 2014 beverage tax changes (pre-post dummy), and unobserved time-invariant household characteristics. *N* = 138,367 household-month obs. (2,842 households).

We observed that nutrient reductions were more pronounced for beverages than for foods for sugar and calories. For example, in Phase 2, there was a 53.6% relative reduction in energy from high-in beverages (-30.6 calories, 95% CI -34.4, -26.8) and a 24.5% relative reduction in total energy from beverages (-24.1 calories, 95% CI -29.2, -19.1), but only a 13.4% relative reduction in energy from high-in foods (-22.6 calories, 95% CI -29.9, -15.4) and a 3.8% relative reduction in total energy from foods (-12.8 calories, 95% CI -25.7, 0.1), with a similar pattern for sugar purchases. Among food purchases, there were significant reductions in both sodium and saturated fat purchases, both among high-in and total food purchases.

In general, the pattern of results in Phase 2 suggested lager changes in purchases than in Phase 1. Relative to the counterfactual based on the pre-policy period, there were larger declines in high-in food, beverage, and total purchases in Phase 2 compared to Phase 1, as well as larger increases in not high-in food, beverage, and total purchases, with mixed results on total food purchases. To further understand how changes in purchases differed between Phase 1 and Phase 2, we examined the underlying intercept and slope changes (S10 Table). Focusing on total nutrients from both high-in and not high-in purchases, all Phase 1 intercept changes for foods, beverages, and foods and beverages combined are negative, together with small positive (or nonsignificant) slope changes for foods and foods and beverages combined, and small negative slope changes for beverages. All Phase 2 intercept changes are positive (but smaller in magnitude than the initial intercept changes) or not statistically significant (sugars from foods and foods and beverages combined). However, these positive intercept changes accommodate for steeper declines in Phase 2 (larger negative slope changes), as can be seen in Fig 2 for foods and beverages overall (especially for energy and sugars). Thus, the Phase 1 changes were mostly driven by large immediate (intercept) changes, while the Phase 2 changes were mostly driven by gradual (slope) changes.

The pattern of results by number of warning labels also differed for foods and beverages (Table 3). In Phase 2, for foods, we observed relative and absolute reductions in calorie purchases only for foods with 2 or more nutrients of concern (-20.3%, -27.1 calories/capita/day, 95% CI -33.4, 20.8), whereas we observed relative and absolute increases in calories purchases for foods with only 1 nutrient of concern (11.8%, 4.2 calories/capita/day, 95% CI 1.9, 6.4). For beverages, we observed large relative reductions in calorie purchases for beverages with 1 nutrient of concern (-51.6%) and very large reductions in calorie purchases for beverages with 2 or more nutrients of concern (-91.8%). However, it is worth noting that the reduction in absolute calories purchased for beverages with 2 or more nutrients of concern was much smaller than for beverages with 1 nutrient of concern (-2.4 calories/capita/day versus -28.0 calories/capita/day, respectively), likely because very few beverages contain high levels of multiple nutrients of concern.

**Table 3:** Estimated average absolute and relative changes in energy (kcal/capita/day) from the no-policy counterfactual by phase and number of excess nutrients of concern.

	Phase 1		Phase 2	
	Abs. diff.	% diff.	Abs. diff.	% diff.
**Total**				
0 excess NOC	**-0.1**	**0.0%**	**15.1**	**7.1%**
	(-5.2, 5.0)	(-2.4, 2.3)	(5.6, 24.6)	(2.3, 11.9)
1 excess NOC	**-16.8**	**-17.6%**	**-22.7**	**-25.7%**
	(-19.6, -14.0)	(-20.1, -15.1)	(-27.2, -18.1)	(-29.6, -21.7)
≥2 excess NOCs	**-23.4**	**-16.9%**	**-29.5**	**-21.6%**
	(-26.9, -19.9)	(19.0, -14.8%)	(-36.0, -23.1)	(-25.4, -17.9)
All	**-40.0**	**-8.8%**	**-36.2**	**-8.3%**
	(-48.5, -31.5)	(-10.5, -7.1)	(-51.8, -20.6)	(-11.6, -5.0)
**Foods**				
0 excess NOC	**-2.0**	**-1.1%**	**10.0**	**5.9%**
	(-6.4, 2.5)	(-3.6, 1.4)	(1.7, 18.3)	(0.7, 11.0)
1 excess NOC	**3.2**	**8.7%**	**4.2**	**11.8%**
	(1.9, 4.6)	(4.8, 12.7)	(1.9, 6.4)	(4.7, 18.8)
≥2 excess NOCs	**-20.8**	**-15.3%**	**-27.1**	**-20.3%**
	(-24.2, -17.4)	(-17.4, -13.2)	(-33.4, -20.8)	(-24.0, -16.5)
All	**-19.6**	**-5.5%**	**-12.8**	**-3.8%**
	(-26.5, -12.7)	(-7.4, -3.7)	(-25.7, 0.1)	(-7.5, -0.1)
**Beverages**				
0 excess NOC	**1.8**	**4.1%**	**5.0**	**11.9%**
	(0.1, 3.5)	(0.0, 8.1)	(2.0, 8.1)	(4.0, 19.9)
1 excess NOC	**-20.9**	**-36.7%**	**-28.0**	**-51.6%**
	(-23.1, -18.6)	(-39.3, -34.1)	(-31.7, -24.3)	(-55.2, -48.0)
≥2 excess NOCs	**-2.6**	**-89.4%**	**-2.4**	**-91.8%**
	(-3.1, -2.1)	(-91.3, -87.4)	(-3.1, -1.7)	(-96.3, -87.3)
All	**-20.9**	**-20.3%**	**-24.1**	**-24.5%**
	(-23.9, -17.9)	(-22.6, -18.0)	(-29.2, -19.1)	(-28.4, -20.5)

Notes: 95% confidence intervals in parentheses. NOC: nutrient of concern (saturated fat, sodium, sugars), plus energy. Adjusted for seasonality (month dummies), household composition (number of household members by age and sex group), SES (4 categories), head of household education level (less than high school, high school, more than high school), region-quarter unemployment rate, number of public holidays in the month, October 2014 beverage tax changes (pre-post dummy), and unobserved time-invariant household characteristics. *N* = 138,367 household-month obs. (2,842 households).

### Differences by SES groups

Compared to their respective counterfactuals, there were few differences in absolute or relative declines in calories from total, food, or beverage purchases by SES (low versus high) in either Phase 1 or Phase 2 of the regulation (Table 4). The exception to this is that for beverages, the relative decline in calories purchased from high-in beverages was greater for high SES versus low SES households in both phases of the law, possibly due to high-SES households’ having lower purchases of high-in beverages during the pre-policy period (S9 Table).

**Table 4:** Estimated average absolute and relative changes in energy (kcal/capita/day) from the no-policy counterfactual by phase, high-in status, and SES.

	Abs. diff.			% diff.		
	High SES	Low SES	Low vs. high	High SES	Low SES	Low vs. high
**Total**						
Phase 1						
High-in	**-40.5**	**-39.8**	**0.7**	**-17.8**	**-16.6**	**1.2**
	(-48.6,-32.5)	(-46.0,-33.6)	(-9.0, 10.5)	(-20.7,-14.9)	(-18.8,-14.5)	(-2.3, 4.6)
Not high-in	**2.8**	**-2.1**	**-4.8**	**1.3**	**-0.9**	**-2.2**
	(-4.4, 9.9)	(-8.4, 4.3)	(-13.7, 4.0)	(-2.1, 4.7)	(-3.7, 1.9)	(-6.3, 1.9)
Total	**-39.5**	**-40.7**	**-1.1**	**-8.9**	**-8.8**	**0.1**
	(-52.6,-26.5)	(-51.1,-30.2)	(-17.1, 14.8)	(-11.5, -6.2)	(-10.9, -6.7)	(-3.2, 3.3)
Phase 2						
High-in	**-54.4**	**-49.9**	**4.5**	**-25.2**	**-21.4**	**3.8**
	(-67.8,-40.9)	(-61.0,-38.8)	(-11.8, 20.7)	(-30.0,-20.4)	(-25.3,-17.6)	(-2.0, 9.6)
Not high-in	**19.9**	**11.8**	**-8.1**	**9.7**	**5.4**	**-4.3**
	(6.4, 33.4)	(0.0, 23.6)	(-24.5, 8.3)	(2.6, 16.8)	(-0.2, 11.0)	(-12.5, 3.9)
Total	**-37.3**	**-35.9**	**1.4**	**-8.9**	**-8.0**	**0.9**
	(-60.6,-14.1)	(-55.2,-16.6)	(-26.8, 29.7)	(-14.0, -3.8)	(-12.0, -4.0)	(-5.2, 7.0)
**Foods**						
Phase 1						
High-in	**-18.2**	**-17.2**	**1.0**	**-10.5**	**-9.7**	**0.9**
	(-24.1,-12.3)	(-22.0,-12.4)	(-6.3, 8.2)	(-13.7, -7.4)	(-12.1, -7.2)	(-2.9, 4.6)
Not high-in	**1.0**	**-3.9**	**-4.9**	**0.7**	**-2.2**	**-2.8**
	(-5.1, 7.1)	(-9.5, 1.7)	(-12.5, 2.6)	(-2.9, 4.2)	(-5.2, 0.9)	(-7.1, 1.5)
Total	**-19.0**	**-20.2**	**-1.2**	**-5.4**	**-5.7**	**-0.2**
	(-29.2, -8.9)	(-28.9,-11.6)	(-13.8, 11.4)	(-8.2, -2.6)	(-7.9, -3.4)	(-3.6, 3.2)
Phase 2						
High-in	**-25.4**	**-20.6**	**4.7**	**-15.5**	**-11.9**	**3.6**
	(-35.7,-15.0)	(-29.6,-11.7)	(-7.9, 17.3)	(-20.9,-10.0)	(-16.5, -7.3)	(-3.0, 10.2)
Not high-in	**16.7**	**5.6**	**-11.0**	**10.2**	**3.2**	**-7.0**
	(5.0, 28.3)	(-4.5, 15.8)	(-25.0, 3.0)	(2.5, 17.9)	(-2.7, 9.1)	(-15.8, 1.8)
Total	**-12.5**	**-13.3**	**-0.8**	**-3.8**	**-3.8**	**0.0**
	(-31.1, 6.1)	(-29.2, 2.7)	(-23.4, 21.9)	(-9.3, 1.6)	(-8.2, 0.6)	(-6.5, 6.5)
**Beverages**						
Phase 1						
High-in	**-26.9**	**-21.8**	**5.1**	**-45.9**	**-36.2**	**9.7**
	(-31.5,-22.4)	(-24.7,-19.0)	(-0.5, 10.7)	(-49.5,-42.3)	(-39.2,-33.1)	(5.1, 14.3)
Not high-in	**1.5**	**2.1**	**0.6**	**3.4**	**4.7**	**1.3**
	(-1.0, 4.0)	(-0.0, 4.2)	(-2.4, 3.6)	(-2.5, 9.3)	(-0.2, 9.6)	(-5.8, 8.4)
Total	**-21.0**	**-20.8**	**0.3**	**-21.4**	**-19.6**	**1.8**
	(-25.9,-16.2)	(-24.5,-17.1)	(-5.7, 6.2)	(-25.1,-17.8)	(-22.4,-16.9)	(-2.7, 6.3)
Phase 2						
High-in	**-34.5**	**-28.3**	**6.2**	**-63.8**	**-48.4**	**15.4**
	(-40.8,-28.2)	(-33.1,-23.5)	(-1.6, 14.0)	(-68.1,-59.4)	(-52.9,-43.9)	(9.4, 21.4)
Not high-in	**3.2**	**6.7**	**3.5**	**7.6**	**15.4**	**7.8**
	(-1.0, 7.3)	(2.8, 10.5)	(-1.6, 8.6)	(-2.9, 18.1)	(5.3, 25.4)	(-5.2, 20.8)
Total	**-25.6**	**-23.2**	**2.3**	**-27.6**	**-22.5**	**5.0**
	(-32.9,-18.2)	(-29.7,-16.8)	(-7.0, 11.7)	(-33.5,-21.7)	(-27.5,-17.6)	(-2.4, 12.4)

Notes: 95% confidence intervals in parentheses. The definition of SES is based on Chile’s Association for Market Research and Public Opinion (*Asociacion de Investigadores de Mercado y Opinion Publica*), which is a measure of SES based on a list of goods and services consumed by the household and the education level of the head of household. Adjusted for seasonality (month dummies), household composition (number of household members by age and sex group), SES (4 categories), region-quarter unemployment rate, number of public holidays in the month, October 2014 beverage tax changes (pre-post dummy), and unobserved time-invariant household characteristics. *N* = 138,367 household-month obs. (2,842 households).

### Food and beverage group adjusted results

Food and beverage subgroup results can be found in Tables 5 and 6, respectively.

**Table 5:** Estimated average absolute and relative changes from the no-policy counterfactual by phase and food group.

	Energy (kcal/capita/day)		Sat. fat (kcal/capita/day)		Sodium (mg/capita/day)		Sugars (kcal/capita/day)	
	Abs. diff.	% diff.	Abs. diff.	% diff.	Abs. diff.	% diff.	Abs. diff.	% diff.
**Phase 1**								
Breakfast cereals	**-1.8**	**-12.1**	**-0.1**	**-15.3**	**-1.5**	**-18.8**	**-0.6**	**-21.4**
	(-2.7, -0.9)	(-17.7, -6.5)	(-0.1, -0.0)	(-21.8, -8.8)	(-2.1, -0.9)	(-24.8,-12.8)	(-0.8, -0.4)	(-27.2,-15.7)
Grain-based desserts	**-2.6**	**-10.8**	**-0.6**	**-11.9**	**-1.7**	**-12.6**	**-0.7**	**-10.6**
	(-3.8, -1.4)	(-15.1, -6.4)	(-0.8, -0.3)	(-16.5, -7.4)	(-2.3, -1.0)	(-17.1, -8.2)	(-1.0, -0.4)	(-14.9, -6.2)
Sweets and non-grain-based desserts	**-1.5**	**-6.3**	**-0.3**	**-10.1**	**0.0**	**-0.1**	**-0.8**	**-5.8**
	(-2.4, -0.5)	(-10.4, -2.2)	(-0.5, -0.1)	(-15.3, -4.8)	(-0.5, 0.4)	(-4.9, 4.7)	(-1.4, -0.2)	(-9.9, -1.7)
Meat, poultry, and meat substitutes	**-2.1**	**-6.4**	**-0.4**	**-4.9**	**-22.8**	**-18.3**	**-0.2**	**-33.0**
	(-3.2, -0.9)	(-9.8, -2.9)	(-0.6, -0.1)	(-8.4, -1.3)	(-27.5,-18.2)	(-21.3,-15.3)	(-0.3, -0.2)	(-36.8,-29.3)
Dairy products and substitutes	**-0.8**	**-3.6**	**-0.3**	**-4.7**	**-1.9**	**-12.1**	**-0.1**	**-1.5**
	(-1.7, 0.2)	(-7.9, 0.7)	(-0.5, 0.0)	(-9.8, 0.4)	(-2.7, -1.2)	(-16.3, -8.0)	(-0.5, 0.2)	(-6.3, 3.2)
Condiments and sauces	**-0.7**	**-2.8**	**-0.2**	**-7.1**	**-13.1**	**-14.7**	**-0.3**	**-7.6**
	(-1.6, 0.3)	(-6.8, 1.3)	(-0.3, -0.0)	(-12.1, -2.0)	(-16.2,-10.0)	(-17.6,-11.8)	(-0.5, -0.2)	(-11.2, -4.0)
Butter and margarine	**-3.4**	**-9.4**	**-1.5**	**-8.4**	**-3.9**	**-10.1**	n/a	n/a
	(-4.8, -2.0)	(-12.9, -5.8)	(-2.2, -0.7)	(-12.2, -4.5)	(-5.4, -2.3)	(-13.8, -6.4)	n/a	n/a
**Phase 2**								
Breakfast cereals	**-1.9**	**-12.5**	**-0.1**	**-9.8**	**-2.6**	**-32.5**	**-0.8**	**-27.8**
	(-3.6, -0.2)	(-22.5, -2.5)	(-0.1, 0.0)	(-22.2, 2.6)	(-3.7, -1.5)	(-41.8,-23.2)	(-1.1, -0.4)	(-37.5,-18.1)
Grain-based desserts	**-2.1**	**-8.4**	**-0.6**	**-11.8**	**-1.6**	**-11.5**	**-0.6**	**-8.8**
	(-4.4, 0.1)	(-16.4, -0.3)	(-1.0, -0.1)	(-20.1, -3.6)	(-2.8, -0.3)	(-19.6, -3.4)	(-1.2, 0.0)	(-16.8, -0.7)
Sweets and non-grain-based desserts	**-0.9**	**-4.2**	**-0.6**	**-21.1**	**0.2**	**1.9**	**-0.4**	**-3.1**
	(-2.5, 0.7)	(-11.4, 3.1)	(-0.9, -0.3)	(-29.3,-12.8)	(-0.6, 0.9)	(-6.6, 10.5)	(-1.3, 0.6)	(-10.4, 4.1)
Meat, poultry, and meat substitutes	**-2.4**	**-7.7**	**-0.5**	**-6.6**	**-20.9**	**-17.6**	**-0.1**	**-18.3**
	(-4.5, -0.3)	(-13.9, -1.5)	(-1.0, 0.0)	(-13.0, -0.3)	(-29.1,-12.7)	(-23.3,-11.8)	(-0.2, -0.1)	(-26.2,-10.4)
Dairy products and substitutes	**-1.5**	**-7.1**	**-0.4**	**-7.9**	**-4.2**	**-26.9**	**-0.2**	**-3.0**
	(-3.3, 0.2)	(-14.9, 0.6)	(-1.0, 0.1)	(-17.2, 1.4)	(-5.5, -2.8)	(-33.4,-20.5)	(-0.9, 0.4)	(-11.5, 5.6)
Condiments and sauces	**1.3**	**5.9**	**0.1**	**4.2**	**-15.0**	**-17.6**	**-0.4**	**-8.5**
	(-0.4, 2.9)	(-2.0, 13.8)	(-0.1, 0.3)	(-5.9, 14.2)	(-20.3, -9.7)	(-22.7,-12.4)	(-0.7, -0.1)	(-14.8, -2.2)
Butter and margarine	**-4.1**	**-11.3**	**-1.5**	**-8.5**	**-8.4**	**-23.0**	n/a	n/a
	(-6.8, -1.5)	(-17.7, -4.8)	(-2.9, -0.1)	(-15.7, -1.3)	(-11.2, -5.7)	(-28.8,-17.1)	n/a	n/a

Notes: Adjusted for seasonality (month dummies), household composition (number of household members by age and sex group), SES (4 categories), head of household education level (less than high school, high school, more than high school), region-quarter unemployment rate, number of public holidays in the month, October 2014 beverage tax changes (pre-post dummy), and unobserved time-invariant household characteristics. *N* = 138,367 household-month obs. (2,842 households).

**Table 6:** Estimated average absolute and relative changes from the no-policy counterfactual by phase and beverage group.

	Energy (kcal/capita/day)		Sugars (kcal/capita/day)	
	Abs. diff.	% diff.	Abs. diff.	% diff.
**Phase 1**				
Sodas	**-8.3**	**-20.9**	**-8.0**	**-20.9**
	(-10.0, -6.6)	(-24.4,-17.5)	(-9.7, -6.4)	(-24.4,-17.4)
Industrialized fruit and vegetable juice	**-5.9**	**-54.3**	**-6.0**	**-58.5**
	(-6.7, -5.1)	(-57.3,-51.3)	(-6.8, -5.2)	(-61.3,-55.7)
Dairy-based beverages and substitutes	**-7.4**	**-16.0**	**-4.4**	**-21.8**
	(-9.2, -5.5)	(-19.3,-12.6)	(-5.2, -3.6)	(-24.8,-18.7)
**Phase 2**				
Sodas	**-9.5**	**-25.9**	**-9.2**	**-25.6**
	(-12.3, -6.6)	(-32.0,-19.9)	(-12.0, -6.3)	(-31.7,-19.5)
Industrialized fruit and vegetable juice	**-8.2**	**-66.4**	**-8.3**	**-70.2**
	(-9.7, -6.7)	(-70.5,-62.3)	(-9.7, -6.8)	(-73.9,-66.6)
Dairy-based beverages and substitutes	**-8.8**	**-19.2**	**-4.9**	**-25.1**
	(-12.1, -5.5)	(-25.1,-13.3)	(-6.3, -3.5)	(-30.5,-19.7)

Notes: Adjusted for seasonality (month dummies), household composition (number of household members by age and sex group), SES (4 categories), head of household education level (less than high school, high school, more than high school), region-quarter unemployment rate, number of public holidays in the month, October 2014 beverage tax changes (pre-post dummy), and unobserved time-invariant household characteristics. *N* = 138,367 household-month obs. (2,842 households).

In general, the pattern of results for food and beverage subgroups in Phase 2 was similar to the overall pattern of results for food and beverage purchases (Tables 5 and 6, respectively). Most food subgroups showed absolute and relative reductions in calories, saturated fat, and sodium purchases, though absolute changes in saturated fat purchases were trivial, and there were no or trivial changes in sugar purchases. One exception to this pattern was condiments and sauces, for which there were no changes in calories or saturated fat and small changes in sugar, but large reduction in sodium purchases, which declined by 17.6% (-15 mg/capita/day, 95% CI -20.3, -9.7). Particularly notable reductions in sodium purchases were also observed for meat, poultry, and meat substitutes (-17.6%, -20.9 mg/capita/day, 95% CI -29.1, -12.7).

Again, similar to the overall beverage results in Phase 2, beverage subgroup purchases showed sizeable absolute and relative reductions in calories and sugar. While purchases of fruit and vegetable drinks showed the largest relative decline in purchases of calories (-66.4%, 95% CI -70.5%, -62.3%) and sugar (-70.2%, 95% CI -73.9%, -66.6%), sodas showed the largest absolute decline in purchases of calories (-9.5 calories/capita/day, 95% CI -12.3, -6.6) and sugar (-9.2 calories/capita/day, 95% CI -12.0, -6.3).

#### Sensitivity analyses

The sensitivity results for total energy can be found in S11 and S12 Tables. The model that allowed for a label rollout period between April and June 2016 led to larger percentage changes, especially for beverages, where the average percentage changes in Phase 1 and Phase 2 go from -20.3% and -24.5% to -23.6% and -29.5%, respectively.

Second, shorter baseline periods led to different patterns of results between foods and beverages. For foods, estimates are broadly consistent across the 36-, 30-, and 24-month baselines, dropping or reversing (and losing statistical significance) with the 18-month baseline. For beverages, shorter baselines were associated with smaller (but statistically significant) estimates. These results are driven by larger changes in the estimates for high-in than for non-high-in foods and beverages (not reported).

The estimates were virtually unchanged without adjusting for the household head’s current education level, when adjusting for household head’s initial education level, and when using the 2-part model for the food and beverage subgroup analyses.

## Discussion

The second phase of Chile’s law of food labeling and advertising continued the warning label, child-directed marketing, and school foods policies, tightened nutrient thresholds for foods subject to these policies, and imposed a daytime ban on all unhealthy food advertisements on television, regardless of audience. After implementation of this second phase, we observed declines in purchases of nutrients of concern from food and drinks carrying the warning label (and thus subject to all regulations), including a 36.8% decline in sugar, 23.0% decline in energy, 21.9% decline in sodium, and 15.7% decline in saturated fat purchased. The declines in high-in products purchased were partially offset by increased purchases from not high-in products. Still, the overall changes resulted in net declines in nutrients of concern purchase ranging from 8.3% to 20.2%.

The pattern of results suggested that, relative to the counterfactual, declines in purchases of high-in foods and drinks were larger in Phase 2 than in Phase 1, as would be expected based on the stricter nutrient thresholds and daytime marketing ban that begin in Phase 2. At the same time, there were also larger increases in purchases of not high-in foods and beverages in Phase 2 than in Phase 1, resulting in a partial attenuation of decreases in total nutrient purchases (e.g., for total calories). It was also interesting to note that in Phase 1, reductions seemed driven by a large immediate drop in nutrients of concern (likely due to reformulation and how the data were linked), whereas in Phase 2, there were larger changes over time, possibly representing behavioral shifts. Future research should investigate the extent to which declines in nutrients of concern were driven by reformulation versus behavioral change, as well as whether these purchasing shifts (i.e., the reduction of high-in foods, increase in not-high-in foods, and moderate decrease in overall nutrients of concern) translate into changes in dietary quality (e.g., type and nutrient density of foods consumed).

We observed differential results for foods versus beverages across policy periods. For example, while declines in total energy were similar for foods and beverages in Phase 1, in Phase 2, the estimated overall decline for beverages was nearly double that of foods. This difference is attributable to a larger drop in high-in purchases and a smaller compensation in not-high-in purchases for beverages compared to foods. With regard to total sugar, there was essentially no change in sugar from food purchases in either phase, whereas the decrease in sugar calories from beverage purchases was sizeable in both Phases due to the large drop in high-in beverages. These results are surprising given that foods were much more affected by stricter nutrient thresholds introduced in Phase 2 (e.g., the sugar threshold for foods went from 22.5 g/100 g in Phase 1 to 15 g/100 g in Phase 2, whereas for beverages, the sugar threshold dropped only from 6 g/100 ml in Phase 1 to 5 g/100 ml in Phase 2).

There are several explanations for the differential results on sugar and energy for foods versus beverages. For one thing, potable water is widely available in Chile, offering a clear substitution for high-in beverage purchases, whereas for foods, presuming people’s purchases of in-store purchases (versus away-from-home foods) remains stable, substitution to other packaged foods (i.e., non-high-in foods) is necessary. A second likely possibility is that beverages are easier to reformulate than are foods, since replacing sugar with nonnutritive sweeteners (NNS) may be more likely to cause issues with texture or taste in food products than in drinks [25–28]. Although data from Phase 2 are not yet available, data on the Chilean food supply from Phase 1 showed that NNS use was most prevalent among beverages before the law as well as the highest absolute increase after the law of any food category [29]. Another evaluation found that, compared to the counterfactual, purchases of NNS in beverages, but not in foods, also increased after Phase 1 of the law [27]. Notably, multiple other countries who have implemented warning label laws after Chile’s have also included a warning for the presence of NNS. It will be important to understand how policy-linked changes in purchases of sugar and NNS compare in these countries as opposed to Chile, which did not include an NNS warning label, particularly for beverage purchases.

A second difference between foods and beverages relates to the number of warning labels on products. Because foods are more nutritionally diverse, they are more likely to be high in multiple nutrients of concern and thus more likely to contain multiple warning labels. We observed that among foods, there were decreases in calories purchased among foods that contained 2 or more nutrients of concern, but increases in calories purchased for foods that contained 1 or no nutrients of concern. This suggests that there is a tendency for consumers to shift purchases away from products with multiple nutrients of concern (i.e., products with more warning labels) toward those with fewer nutrients of concern (i.e., products with fewer warning labels), either through changes in consumer behavior (selecting a product with 1 versus 2 or more labels) or reformulation (companies reducing the nutrient content so as to reduce the total number of warning labels on the package). In contrast, among beverages, it is much more common for products to carry only 1 nutrient of concern, usually on sugar content. Consequently, we observed calorie reductions for purchases of products with 1 nutrient of concern. Taken together, the results suggest an overall shift from products with more versus fewer nutrients of concern: For foods, the shift is from products with multiple nutrients of concern to those with fewer nutrients of concern, whereas for beverages, the shift is from products with a single nutrient of concern to those with no nutrients of concern.

A third difference between foods and beverages is that the policies were associated with important declines in sodium purchases from foods, whereas for beverages, we did not analyze sodium because so few products were high in them. Purchases of sodium from high-in foods declined by approximately 74 mg/sodium/capita/day, or a nearly 20% relative reduction, and these decreases led to a net reduction of 64 mg/capita/day of total sodium purchases from foods. Sodium reductions occurred across every food group except sweets and other desserts and were particularly sizeable for meat, poultry, and meat substitutes, condiments and sauces, and butter and margarine. These results are encouraging, considering that Chile is one of only 5% of the 194 WHO member countries to achieve the highest score for sodium reduction policies, which includes having at least 2 mandatory policies, all WHO sodium-related “best buy” practices, and sodium declarations on packaged foods [30]. Our results suggest that such policies lead to reductions in sodium purchases, though more research is needed to understand how these changes translate to achieving dietary targets for sodium in the Chilean population as well as subsequent sodium-related health benefits such as reduction of high blood pressure and cardiovascular disease. Overall, the results across sugar, sodium, and saturated fat highlight the need to include both foods and beverages in policies in order to reduce nutrients of concern.

The results that low-SES households reduced purchases of nutrients of concern after policy implementation were consistent with other data from our evaluation. Our focus group data found that low-SES parents paid attention to, understood, and used the warning labels [8,31]. In addition, a recent study found that during Phase 1 of the policy implementation, there were no price changes for high-in or not-high in foods [32]. Such price changes, for example, if not high-in-food prices increased as the result of companies passing on the cost of reformulation, could have dampened the effect among low-SES households who tend to be more price sensitive. Instead, the lack of price changes likely increased low-SES parents’ ability to attend to and incorporate information from the warning labels into their decision-making. However, a key limitation of our current study is that it did not include data after June 2019, a period which was marked by Chilean social unrest, food supply chain volatility, global food price inflation, and the COVID-19 pandemic and lockdowns, events that could have blunted the effect of the policies in low-SES households. Our qualitative data from 2021 suggested that even though the labels helped parents understand which foods were healthy versus unhealthy, low-SES parents were struggling to choose the healthy options due to cost [31]. Thus, more research is needed to understand the ways in which marketing, labeling, and school foods policies intersect with food prices, particularly during times of economic instability and global food price increases. Moreover, it is important to recognize that price remains a top driver of food choice, particularly for income-constrained households, and marketing and labeling policies to disincentivize unhealthy food choices may have limited effect if not implemented alongside policies that increase affordability or availability of healthier food choices.

### Limitations

This study had important limitations. One limitation is that the educational attainment of households increased over time more so than did the national sample, likely due to increases in education of panelists and the higher education of new panelists who joined the sample over time. Although education is included in our analytical models, we cannot rule out the possibility that increased education may have partially contributed to reductions in purchases of high-in foods over the study period.

Another limitation is that while the construction of a counterfactual allowed us to compare observed purchases in the post-policy period to what would have happened in the absence of the policy, decisions about how to create the counterfactual are somewhat arbitrary and can influence results. For example, we did not have nutrition data collected from supermarkets for 2013 to 2014 and thus relied on a combination of 2015 nutrition data for those years and data from the Mintel New Products Database. While some data suggest that the nutrient content of the Chilean food supply was stable during 2015 to 2016 [33] (the year prior to the law’s implementation), we cannot be sure if this was true in 2013 to 2014, which may have also affected the counterfactual. Sensitivity analyses revealed that results were sensitive to the length of the baseline period, with the shortest time period (18-month baseline) associated with smaller (and no longer statistically significant) reductions in total calories from foods, and shorter time periods (30-, 24-, and 18-month baselines) associated with gradually smaller (albeit statistically significant) reductions in total calories from beverages. Because these differences could reflect a number of factors (actual trends in food purchases but also potential changes in data collection procedures and sociodemographic characteristics of the panel over time, as well as the lack of quality nutrient data before 2015), it is impossible to say for certain the extent to which the shorter versus longer baseline period should be preferred.

More broadly, the collection of nutrition data occurred only once per year, and we updated the linkage between nutrition data and purchases data once annually, which can influence the timing for which reformulation affects the nutrient content of purchases (either earlier or later than the product was really reformulated and available for purchase). This may have resulted in an overestimation of effect, since we used data collected later to link to earlier purchases in the same policy window (e.g., nutrition information from Q1 of 2019 was preferentially applied to purchases made from July 2018 to December 31, 2018). We also provide a more in-depth comparison of current findings with our team’s previous findings in S2 Appendix [10].

Lastly, we could not evaluate changes in food purchases during Phase 3 of the Chilean law as originally planned. Phase 3 was implemented in June of 2019. In the following year, the food supply and food purchasing behaviors were severely impacted by national protests in October to December 2019 and lockdowns due to the onset of the COVID-19 pandemic, making it impossible to analyze purchasing trends during this period. However, the results in this study are likely similar to what we would have observed during Phase 3. Although the nutrient thresholds became slightly stricter in Phase 2, there was only a 2% increase in the prevalence of high-in products, and there were no other substantive policy changes during this time. Of course, we cannot rule out other changes that could have occurred during Phase 3 (e.g., more consumer fatigue), but generally, the results presented here for Phase 2 are likely to represent the full implementation of the Chilean law.

### Conclusions

After the second phase of implementation of the Chilean Law of Food Labeling and Advertising, compared to a counterfactual constructed from trends during a 36-month pre-policy timeframe, we observed significant decreases in high-in purchases of foods and beverages during Phase 2, including a relative 36.8% reduction in sugar, a 23.0% relative reduction in energy, a 21.9% relative reduction in sodium, and a 15.7% relative reduction in saturated fat. Decreases were partially offset by increases in not high-in purchases, but there was still a significant decrease in total nutrients of concern purchased during Phase 2. Moreover, the pattern of results suggested bigger declines in purchases of high-in foods and bigger increases in purchases of not high-in foods in Phase 2 than in Phase 1, suggesting sustained or even increasing effects of the policy over time. Reductions in sugar and energy were driven by beverage purchases, whereas reductions in sodium and saturated fat were driven by foods. The pattern of declines in purchases was similar for households of lower versus higher SES. However, results must be interpreted with caution due to the sensitivity of findings to different model specifications, such as the length of the pre-policy timeframe that was used to create the post-policy counterfactual. Future research will be needed to replicate these findings as well as to understand whether and how purchasing shifts translated to changes in dietary quality and health.

## Supporting information

S1 Table Sample exclusion criteria and number and percent of observations per exclusion criterion at the purchase and household-month levels. Notes: 1. In February 2017, Kantar Worldpanel (KWP) switched from calendar months to custom production periods running from the last week of a month to the last week of the following month to compile purchase records. As a result, purchase records were allocated to both the first and second production periods of 2017. To identify the duplicated records, KWP modified their purchase date year to 1997. Since we aggregated data at the household-calendar month level, these records were removed so as not to double-count them. 2. Bulk products were excluded due to lack of product-specific information (e.g., weight, barcode) allowing for linkage to nutrition facts panel data. 3. Across data years, between 93.4% and 95.7% of all zero-price non-bulk purchases were reported as gifts. As a proportion of total non-bulk purchases, the prevalence of these purchases has been relatively constant, ranging from 1.5% to 3.4% by data year. 4. Including purchases of the only product that was recategorized as a snack upon the introduction of this category, based on a search of snack barcodes in other categories. 5. 5,252,524 after aggregating at the household-day-product level. After all exclusions were implemented, a further 453 household-day-product observations were dropped because the calendar month of the purchase was the month preceding the household’s first production period, as households newly added to the panel after the production period change mentioned in Note 1 begin contributing data at the end of their first calendar month rather than at the start. 6. Another 24 household-month observations were excluded from the estimation sample because of missing administrative region, which meant that the regional unemployment rate, which is included in the models, was missing for these observations.
(DOCX)

S2 Table Comparison of the 2017 weighted sample to 2017 population estimates for urban households from the Census or the CASEN survey. Sources: Census 2017 and CASEN 2017. CASEN (Chile Encuesta Nacional de Caracterización Socio-económica) is a nationally representative household survey conducted by Chile’s statistics office to provide official statistics on a range of topics. Notes: 1. Observations are at the household-month level. 2. Source: CASEN. 3. Source: Census. The number of urban households underlying the Census percentages were calculated by multiplying the total number of households by the proportion of individuals living in urban areas by region. CASEN estimates show that household size is similar in urban and rural areas nationwide (means [standard errors]: 3.07 [0.01] and 3.08 [0.02], respectively), so the overall proportion of individuals living in urban areas is a good approximation of the overall proportion of urban households. Although there may be regional differences in household size between urban and rural areas, CASEN estimates also show that there is little variation in household size by broad economic region among rural households. 4. Source: CASEN. CASEN percentages do not sum to 100 because of missing values. 5. Source: CASEN. Main shopper’s age in the weighted sample, head of household’s age in CASEN.
(DOCX)

S3 Table Food and beverage categories included and their aggregate expenditure shares. Note: Asterisks indicate categories that had ≥5% share of expenditures in food or in beverages and were included in the food and beverage subgroup analyses.
(DOCX)

S4 Table Nutrient thresholds and implementation dates of the Chilean Labeling and Advertising Law. (DOCX)

S5 Table Nutrition Facts Panel source by linking period and match type. Note: Direct matches are 1:1 barcode matches between the purchase and nutrition facts panel data. Products matched on other characteristics in the absence of a direct match are indirect matches. INTA, Institute of Nutrition and Food Technology; Mintel, Mintel Global New Products Database (Americas).
(DOCX)

S6 Table Changes in the distribution of households by household head’s current and initial education levels (% of households) in the weighted sample compared to national estimates for the urban population from CASEN. Notes: Initial education level: education level in 2013 or enrollment year for new participants. CASEN: Chile Encuesta Nacional de Caracterización Socio-económica. ^*e*^: extrapolated from the nearest 2 estimates assuming a linear change. CASEN totals do not necessarily sum to 100 due to missing values.
(DOCX)

S7 Table Raw percentages of products with 0, 1, and 2 or more excess nutrients of concern (NOC) (Phase 3 limits) by period (July 2013-June 2019). Notes: NOC are sugar, saturated fat, sodium, and energy. As a result of the nutrition facts panel data linking protocol, the number of excess NOCs a product has may vary within a given period (multiple years of nutrition facts panel data used in the pre-policy and Phase 1 periods).
(DOCX)

S8 Table Unadjusted weighted means (standard deviation) in food and beverage purchases by July-June period. (DOCX)

S9 Table Unadjusted weighted mean (standard deviation) nutrient content of food and beverage purchases by policy period and socioeconomic status (SES). (DOCX)

S10 Table Impact model parameter estimates (percentage changes). Notes: *** *p* < .01, ** *p* < .05, * *p* < .1. Standard errors in parentheses. The July 2018 (Phase 2) estimates are relative to the Phase 1 trend; for example, a nonzero July 2018 slope change indicates a change in the slope of the trend from phase 1. Adjusted for seasonality (month dummies), household composition (number of household members by age and sex group), SES (4 categories), head of household education level (less than high school, high school, more than high school), region-quarter unemployment rate, number of public holidays in the month, October 2014 beverage tax changes (pre-post dummy), and unobserved time-invariant household characteristics. *N* = 138,367 household-month obs. (2,842 households).
(DOCX)

S11 Table Sensitivity results (average percentage changes in total energy). Notes: *** *p* < .01, ** *p* < .05, * *p* < .1. Standard errors in parentheses. The AICs and BICs are divided by 100 and rounded to the nearest integer to facilitate comparisons (omitted in (3)-(5) because each uses a different estimation dataset). All estimates are CRE Poisson and adjusted for seasonality (month dummies), household composition (number of household members by age and sex group), SES (4 categories), head of household education level (less than high school, high school, more than high school), region-quarter unemployment rate, number of public holidays in the month, October 2014 beverage tax changes (pre-post dummy), and unobserved time-invariant household characteristics (unless otherwise noted).
(DOCX)

S12 Table One-part vs. two-part model estimates for total energy by food and beverage subgroup (average difference with the counterfactual). Note: Standard errors in parentheses. Adjusted for seasonality (month dummies), household composition (number of household members by age and sex group), SES (4 categories), head of household education level (less than high school, high school, more than high school), region-quarter unemployment rate, number of public holidays in the month, October 2014 beverage tax changes (pre-post dummy), and unobserved time-invariant household characteristics. *N* = 138,367 household-month obs. (2,842 households).
(DOCX)

S1 Fig Food purchases data linked to nutrition data from INTA (Institute of Nutrition and Food Technology) and Mintel by year, according to Chilean policy period. (DOCX)

S1 Appendix Models and effects. (DOCX)

S2 Appendix Differences between papers. (DOCX)

S1 STROBE Statement Checklist of items that should be included in reports of observational studies. (DOCX)

## Abbreviations

AIM: Asociacion de Investigadores de Mercado y Opinion Publica de Chile
CASEN: Encuesta de Caracterización Socioeconómica
COVID-19: Coronavirus Disease 2019
INTA: Institute of Nutrition and Food Technology
NFP: nutrition facts panel
NNS: nonnutritive sweetener
SES: socioeconomic status

## References

[1] Popkin BM, Barquera S, Corvalan C, Hofman KJ, Monteiro C, Ng SW, et al. Towards unified and impactful policies to reduce ultra-processed food consumption and promote healthier eating. Lancet Diabetes Endocrinol. 2021;9(7):462–470. doi: 10.1016/S2213-8587(21)00078-4 33865500 PMC8217149

[2] Reyes M, Smith Taillie L, Popkin B, Kanter R, Vandevijvere S, Corvalán C. Changes in the amount of nutrient of packaged foods and beverages after the initial implementation of the Chilean Law of Food Labelling and Advertising: A nonexperimental prospective study. PLoS Med. 2020;17(7):e1003220. doi: 10.1371/journal.pmed.1003220 32722710 PMC7386631

[3] Correa T, Reyes M, Taillie LS, Corvalan C, Dillman Carpentier FR. Food Advertising on Television Before and After a National Unhealthy Food Marketing Regulation in Chile, 2016–2017. Am J Public Health. 2020;110(7):1054–1059. doi: 10.2105/AJPH.2020.305658 32437274 PMC7287518

[4] Dillman Carpentier FR, Correa T, Reyes M, Taillie LS. Evaluating the impact of Chile’s marketing regulation of unhealthy foods and beverages: pre-school and adolescent children’s changes in exposure to food advertising on television. Public Health Nutr. 2020;23(4):747–755. doi: 10.1017/S1368980019003355 31822317 PMC7060093

[5] Jensen ML, Carpentier FD, Adair L, Corvalan C, Popkin BM, Taillie LS. Examining Chile’s unique food marketing policy: TV advertising and dietary intake in preschool children, a pre- and post- policy study. Pediatr Obes. 2021;16(4):e12735. doi: 10.1111/ijpo.12735 33105522 PMC8073213

[6] Jensen ML, Dillman Carpentier FR, Adair L, Corvalán C, Popkin BM, Taillie LS. TV advertising and dietary intake in adolescents: a pre-and post-study of Chile’s food marketing policy. Int J Behav Nutr Phys Act. 2021;18:1–11. 33947436 10.1186/s12966-021-01126-7 PMC8097821

[7] Mediano Stoltze F, Reyes M, Smith TL, Correa T, Corvalan C, Carpentier FRD. Prevalence of Child-Directed Marketing on Breakfast Cereal Packages before and after Chile’s Food Marketing Law: A Pre- and Post-Quantitative Content Analysis. Int J Environ Res Public Health. 2019;16(22):4501. doi: 10.3390/ijerph16224501 31731577 PMC6888536

[8] Correa T, Fierro C, Reyes M, Carpentier FRD, Taillie LS, Corvalan C. Responses to the Chilean law of food labeling and advertising: exploring knowledge, perceptions and behaviors of mothers of young children. Int J Behav Nutr Phys Act. 2019;16(1):21. doi: 10.1186/s12966-019-0781-x 30760273 PMC6375144

[9] Taillie LS, Reyes M, Colchero MA, Popkin B, Corvalán C. An evaluation of Chile’s Law of Food Labeling and Advertising on sugar-sweetened beverage purchases from 2015 to 2017: A before-and-after study. PLoS Med. 2020;17(2):e1003015. doi: 10.1371/journal.pmed.1003015 32045424 PMC7012389

[10] Taillie LS, Bercholz M, Popkin B, Reyes M, Colchero MA, Corvalán C. Changes in food purchases after the Chilean policies on food labelling, marketing, and sales in schools: a before and after study. Lancet Planet Health. 2021;5(8):e526–e533. doi: 10.1016/S2542-5196(21)00172-8 34390670 PMC8364623

[11] Barahona N, Otero C, Otero S, Kim J. Equilibrium Effects of Food Labeling Policies. Econometrica. 2023;91(3):839–868.

[12] Massri C, Sutherland S, Källestål C, Peña S. Impact of the food-labeling and advertising law banning competitive food and beverages in Chilean public schools, 2014–2016. Am J Public Health. 2019;109(9):1249–1254. doi: 10.2105/AJPH.2019.305159 31318604 PMC6687276

[13] Fretes G, Corvalán C, Reyes M, Taillie LS, Economos CD, Wilson NL, Cash SB. Changes in children’s and adolescents’ dietary intake after the implementation of Chile’s law of food labeling, advertising and sales in schools: a longitudinal study. Int J Behav Nutr Phys Act. 2023;20(1):40. doi: 10.1186/s12966-023-01445-x 37016430 PMC10074676

[14] Colchero MA, Rivera-Dommarco J, Popkin B, Ng SW. In Mexico, evidence of sustained consumer response two years after implementing a sugar-sweetened beverage tax. Health Aff (Millwood). 2017;36(3):564–571. doi: 10.1377/hlthaff.2016.1231 28228484 PMC5442881

[15] Powell LM, Leider J. Impact of a sugar-sweetened beverage tax two-year post-tax implementation in Seattle, Washington, United States J Public Health Policy. 2021;42:574–588. doi: 10.1057/s41271-021-00308-8 34732842

[16] Bleich SN, Dunn CG, Soto MJ, Yan J, Gibson LA, Lawman HG, et al. Association of a sweetened beverage tax with purchases of beverages and high-sugar foods at independent stores in Philadelphia. JAMA Netw Open. 2021;4(6):e2113527. doi: 10.1001/jamanetworkopen.2021.13527 34129022 PMC8207239

[17] Dillman Carpentier FR, Mediano Stoltze F, Reyes M, Taillie LS, Corvalán C, Correa T. Restricting child-directed ads is effective, but adding a time-based ban is better: evaluating a multi-phase regulation to protect children from unhealthy food marketing on television. Int J Behav Nutr Phys Act. 2023;20(1):62. doi: 10.1186/s12966-023-01454-w 37231508 PMC10214667

[18] Shrestha A, Cullerton K, White KM, Mays J, Sendall M. Impact of front-of-pack nutrition labelling in consumer understanding and use across socio-economic status: A systematic review. Appetite. 2023;187:106587. doi: 10.1016/j.appet.2023.106587 37169260

[19] Hough G, Sosa M. Food choice in low income populations–A review. Food Qual Prefer. 2015;40:334–342.

[20] Chile IcE. ENCUESTA NACIONAL DE EMPLEO—ENE Santiago: Instituto cione Estadasticas: Chile. 2020. https://webanterior.ine.cl/estadisticas/laborales/ene.

[21] Kanter R, Reyes M, Corvalán C. Photographic methods for measuring packaged food and beverage products in supermarkets. Curr Dev Nutr. 2017;1(10):e001016. doi: 10.3945/cdn.117.001016 29955678 PMC5998779

[22] Corvalán C, Reyes M, Garmendia ML, Uauy R. Structural responses to the obesity and non-communicable diseases epidemic: Update on the Chilean law of food labelling and advertising. Obes Rev. 2019;20(3):367–374. doi: 10.1111/obr.12802 30549191

[23] Barahona N, Otero C, Otero S, Kim J. Single-Threshold Food Labeling Policies. 2023. https://sebotero.github.io/papers/foodlabels_policy.pdf.

[24] Araya S, Elberg A, Noton C, Schwartz D. Identifying Food Labeling Effects on Consumer Behavior. Mar Sci. 2022;41(5):982–1003.

[25] Rebolledo N, Reyes M, Popkin BM, Adair L, Avery CL, Corvalán C, et al. Changes in nonnutritive sweetener intake in a cohort of preschoolers after the implementation of Chile’s Law of Food Labelling and Advertising. Pediatr Obes. 2022;17(7):e12895. doi: 10.1111/ijpo.12895 35088571

[26] Rebolledo N, Bercholz M, Corvalán C, Ng SW, Taillie LS. Did the sweetness of beverages change with the Chilean Food Labeling and Marketing Law? A before and after study. Front Nutr. 2022;9:1043665. doi: 10.3389/fnut.2022.1043665 36386952 PMC9650246

[27] Rebolledo N, Bercholz M, Adair L, Corvalán C, Ng SW, Taillie LS. Sweetener Purchases in Chile before and after Implementing a Policy for Food Labeling, Marketing, and Sales in Schools. Curr Dev Nutr. 2023;7(2):100016. doi: 10.1016/j.cdnut.2022.100016 37180088 PMC10111599

[28] Fuentealba NR, Reyes M, Corvalan C, Popkin B, Taillie LS. Do Sugary Drink Policies Increase Purchases of Non-Calorically Sweetened Beverages? Evidence from Chile. Curr Dev Nutr. 2020;4(Supplement_2):1478.

[29] Zancheta Ricardo C, Corvalán C, Smith Taillie L, Quitral V, Reyes M. Changes in the use of non-nutritive sweeteners in the Chilean food and beverage supply after the implementation of the food labeling and advertising law. Front Nutr. 2021;8:773450. doi: 10.3389/fnut.2021.773450 34859036 PMC8630583

[30] World Health Organization. WHO Global Report on Sodium Intake Reduction. 2023.

[31] Correa T, Fierro C, Reyes M, Taillie LS, Carpentier FRD, Corvalán C. Why Don’t You [Government] Help Us Make Healthier Foods More Affordable Instead of Bombarding Us with Labels? Maternal Knowledge, Perceptions, and Practices after Full Implementation of the Chilean Food Labelling Law. Int J Environ Res Public Health. 2022;19(8):4547. doi: 10.3390/ijerph19084547 35457415 PMC9025178

[32] Paraje G, de Oca DM, Corvalán C, Popkin BM. Evolution of food and beverage prices after the front-of-package labelling regulations in Chile. BMJ Glob Health. 2023;8(7):e011312. doi: 10.1136/bmjgh-2022-011312 37400119 PMC10335503

[33] Kanter R, Reyes M, Vandevijvere S, Swinburn B, Corvalán C. Anticipatory effects of the implementation of the Chilean Law of Food Labeling and Advertising on food and beverage product reformulation. Obes Rev. 2019;20(Suppl 2):129–140. doi: 10.1111/obr.12870 31245920

