# Supplementary material for: Decreases in purchases of energy, sodium, sugar, and saturated fat 3 years after implementation of the Chilean food labeling and marketing law: An interrupted time series analysis
Source: PLoS Med. 2024 Sep 27;21(9):e1004463. doi: 10.1371/journal.pmed.1004463 (PMC11432892; doi:10.1371/journal.pmed.1004463)
Supplement: S1 Table — Notes: 1. In February 2017, Kantar Worldpanel (KWP) switched from calendar months to custom production periods running from the last week of a month to the last week of the following month to compile purchase records. As a result, purchase records were allocated to both the first and second production periods of 2017. To identify the duplicated records, KWP modified their purchase date year to 1997. Since we aggregated data at the household-calendar month level, these records were removed so as not to double-count them. 2. Bulk products were excluded due to lack of product-specific information (e.g., weight, barcode) allowing for linkage to nutrition facts panel data. 3. Across data years, between 93.4% and 95.7% of all zero-price non-bulk purchases were reported as gifts. As a proportion of total non-bulk purchases, the prevalence of these purchases has been relatively constant, ranging from 1.5% to 3.4% by data year. 4. Including purchases of the only product that was recategorized as a snack upon the introduction of this category, based on a search of snack barcodes in other categories. 5. 5,252,524 after aggregating at the household-day-product level. After all exclusions were implemented, a further 453 household-day-product observations were dropped because the calendar month of the purchase was the month preceding the household’s first production period, as households newly added to the panel after the production period change mentioned in Note 1 begin contributing data at the end of their first calendar month rather than at the start. 6. Another 24 household-month observations were excluded from the estimation sample because of missing administrative region, which meant that the regional unemployment rate, which is included in the models, was missing for these observations. (DOCX) [file pmed.1004463.s001.docx]

S1 Table. Sample exclusion criteria and number and percent of observations per exclusion criterion at the purchase and household-month levels.

|  | n | % |
| --- | --- | --- |
| **Observations at the purchase level** |  |  |
| Purchase date year is 1997^1^ | 19,399 | 0 |
| Bulk purchase^2^ | 1,930,179 | 24 |
| Zero price^3^ | 156,481 | 2 |
| Zero quantity | 1 | 0 |
| No nutrition facts data | 637,311 | 8 |
| Kantar categories excluded | 636,831 | 8 |
| Tea and infusions | 161,875 | 2 |
| Baby food products | 14,677 | 0 |
| Raw sugar | 187,583 | 2 |
| Non-caloric sweeteners | 34,671 | 0 |
| Oil | 238,025 | 3 |
| Other | 480 | 0 |
| Additional baby food and formula products | 11,601 | 0 |
| Kantar categories with incomplete data over study period | 117,812 | 1 |
| Cereal bars | 1,243 | 0 |
| Minced meat | 2,046 | 0 |
| Condensed milk | 25,340 | 0 |
| Snacks^4^ | 89,183 | 1 |
| Total excluded (any of the above) | 2,828,700 | 35 |
| Total remaining^5^ | 5,345,327 | 65 |
| Grand total | 8,174,027 | 100 |
| **Observations at the household-month level** |  |  |
| Zero total energy across all food and beverage purchases | 9 | 0 |
| Missing household size | 25 | 0 |
| Total excluded (any of the above) | 34 | 0 |
| Total remaining^6^ | 149,799 | 100 |
| Grand total | 149,833 | 100 |

Notes:

1. In February 2017, Kantar Worldpanel (KWP) switched from calendar months to custom production periods running from the last week of a month to the last week of the following month to compile purchase records. As a result, purchase records were allocated to both the first and second production periods of 2017. To identify the duplicated records, KWP modified their purchase date year to 1997. Since we aggregated data at the household-calendar month level, these records were removed so as not to double-count them.
2. Bulk products were excluded due to lack of product-specific information (e.g., weight, barcode) allowing for linkage to nutrition facts panel data.
3. Across data years, between 93.4% and 95.7% of all zero-price non-bulk purchases were reported as gifts. As a proportion of total non-bulk purchases, the prevalence of these purchases has been relatively constant, ranging from 1.5% to 3.4% by data year.
4. Including purchases of the only product that was recategorized as a snack upon the introduction of this category, based on a search of snack barcodes in other categories.
5. 5,252,524 after aggregating at the household-day-product level. After all exclusions were implemented, a further 453 household-day-product observations were dropped because the calendar month of the purchase was the month preceding the household’s first production period, as households newly added to the panel after the production period change mentioned in Note 1 begin contributing data at the end of their first calendar month rather than at the start.
6. Another 24 household-month observations were excluded from the estimation sample because of missing administrative region, which meant that the regional unemployment rate, which is included in the models, was missing for these observations.
