# Supplementary material for: Decreases in purchases of energy, sodium, sugar, and saturated fat 3 years after implementation of the Chilean food labeling and marketing law: An interrupted time series analysis
Source: PLoS Med. 2024 Sep 27;21(9):e1004463. doi: 10.1371/journal.pmed.1004463 (PMC11432892; doi:10.1371/journal.pmed.1004463)
Supplement: S2 Table — Sources: Census 2017 and CASEN 2017. CASEN (Chile Encuesta Nacional de Caracterización Socio-económica) is a nationally representative household survey conducted by Chile’s statistics office to provide official statistics on a range of topics. Notes: 1. Observations are at the household-month level. 2. Source: CASEN. 3. Source: Census. The number of urban households underlying the Census percentages were calculated by multiplying the total number of households by the proportion of individuals living in urban areas by region. CASEN estimates show that household size is similar in urban and rural areas nationwide (means [standard errors]: 3.07 [0.01] and 3.08 [0.02], respectively), so the overall proportion of individuals living in urban areas is a good approximation of the overall proportion of urban households. Although there may be regional differences in household size between urban and rural areas, CASEN estimates also show that there is little variation in household size by broad economic region among rural households. 4. Source: CASEN. CASEN percentages do not sum to 100 because of missing values. 5. Source: CASEN. Main shopper’s age in the weighted sample, head of household’s age in CASEN. (DOCX) [file pmed.1004463.s002.docx]

S2 Table. Comparison of the 2017 weighted sample to 2017 population estimates for urban households from the Census or the CASEN survey.

|  | Weighted sample^1^ | Census or CASEN |
| --- | --- | --- |
| Household size^2^ (mean) | 4.3 | 3.1 |
| Region^3^ (%) |  |  |
| Arica y Parinacota | 2.0 | 1.2 |
| Tarapaca | 1.9 | 1.9 |
| Antofagasta | 3.7 | 3.3 |
| Atacama | 1.3 | 1.6 |
| Coquimbo | 4.2 | 3.9 |
| Valparaiso | 11.0 | 11.2 |
| Metropolitana de Santiago | 48.3 | 43.6 |
| Liberator General Bernardo O’Higgins | 3.5 | 4.5 |
| Maule | 5.2 | 5.2 |
| Nuble | (none) | 2.3 |
| Biobio | 10.4 | 9.1 |
| La Araucania | 3.6 | 4.6 |
| Los Rios | 1.9 | 1.9 |
| Los Lagos | 3.0 | 4.1 |
| Aysendel General Carlos Ibanez del Campo | (none) | 0.6 |
| Magallanes y de la antartica Chilena | (none) | 1.0 |
| Head of household’s education level^4^ (%) |  |  |
| Primary or less, including incomplete secondary | 31.0 | 40.0 |
| Secondary, including incomplete tertiary | 42.0 | 36.1 |
| Tertiary | 27.0 | 23.3 |
| Main shopper and head of household’s age group^5^ (%) |  |  |
| 29 or younger | 7.8 | 8.5 |
| 30-44 | 34.1 | 25.0 |
| 45-59 | 32.7 | 31.1 |
| 60 or older | 25.5 | 35.4 |

Sources: Census 2017 and CASEN 2017. CASEN (Encuesta de Caracterización Socioeconómica Nacional) is a nationally representative household survey conducted by Chile’s statistics office to provide official statistics on a range of topics.

Notes:

1. Observations are at the household-month level.
2. Source: CASEN.
3. Source: Census. The number of urban households underlying the Census percentages were calculated by multiplying the total number of households by the proportion of individuals living in urban areas by region. CASEN estimates show that household size is similar in urban and rural areas nationwide (means [standard errors]: 3.07 [0.01] and 3.08 [0.02], respectively), so the overall proportion of individuals living in urban areas is a good approximation of the overall proportion of urban households. Although there may be regional differences in household size between urban and rural areas, CASEN estimates also show that there is little variation in household size by broad economic region among rural households.
4. Source: CASEN. CASEN percentages do not sum to 100 because of missing values.
5. Source: CASEN. Main shopper’s age in the weighted sample, head of household’s age in CASEN.
