# Supplementary material for: Decreases in purchases of energy, sodium, sugar, and saturated fat 3 years after implementation of the Chilean food labeling and marketing law: An interrupted time series analysis
Source: PLoS Med. 2024 Sep 27;21(9):e1004463. doi: 10.1371/journal.pmed.1004463 (PMC11432892; doi:10.1371/journal.pmed.1004463)
Supplement: S3 Table — Note: Asterisks indicate categories that had ≥5% share of expenditures in food or in beverages and were included in the food and beverage subgroup analyses. (DOCX) [file pmed.1004463.s003.docx]

S3 Table. Food and beverage categories included and their aggregate expenditure shares.

| **Category** | **% of food exp.** | **% of bev. exp.** | **% of total exp.** |
| --- | --- | --- | --- |
| Cereal-based foods | 22.1 |  | 13.6 |
| Meat, poultry and meat substitutes* | 15.7 |  | 9.6 |
| Dairy products and dairy substitutes* | 13.1 |  | 8.0 |
| Sweets and non-grain-based desserts* | 12.2 |  | 7.5 |
| Condiments and sauces* | 10.2 |  | 6.3 |
| Oils and fats* | 8.1 |  | 5.0 |
| Grain-based desserts* | 7.2 |  | 4.4 |
| Breakfast cereals* | 5.0 |  | 3.1 |
| Soups | 3.3 |  | 2.0 |
| Snacks | 2.0 |  | 1.2 |
| Traditional mixed dishes | 0.5 |  | 0.3 |
| Salt and seasoning | 0.3 |  | 0.2 |
| Fruits, vegetables and mushrooms | 0.2 |  | 0.1 |
| Fish and seafood | 0.0 |  | 0.0 |
| Total food | 100.0 |  | 61.4 |
| Dairy-based beverages and dairy substitutes* |  | 37.5 | 14.5 |
| Sodas* |  | 33.4 | 13.0 |
| Industrialized, fruit and vegetable juice* |  | 9.9 | 3.8 |
| Water |  | 9.7 | 3.8 |
| Coffee and tea |  | 9.3 | 3.6 |
| 100% fruit/vegetable juice |  | 0.1 | 0.0 |
| Sports and energy drinks |  | 0.0 | 0.0 |
| Total beverages |  | 100.0 | 38.7 |
| Grand total |  |  | 100.0 |

Note: asterisks indicate categories that had ≥ 5% share of expenditures in food or in beverages and were included in the food and beverage sub-group analyses.
