# Supplementary material for: Decreases in purchases of energy, sodium, sugar, and saturated fat 3 years after implementation of the Chilean food labeling and marketing law: An interrupted time series analysis
Source: PLoS Med. 2024 Sep 27;21(9):e1004463. doi: 10.1371/journal.pmed.1004463 (PMC11432892; doi:10.1371/journal.pmed.1004463)
Supplement: S4 Table — (DOCX) [file pmed.1004463.s004.docx]

S4 Table. Nutrient thresholds and implementation dates of the Chilean Labeling and Advertising Law.

|  | **26 June 2016** | **26 June 2018** | **26 June 2019** |
| --- | --- | --- | --- |
| **Solid food (per 100 g)** |  |  |  |
| Energy (kcal) | 350 | 300 | 275 |
| Sodium (mg) | 800 | 500 | 400 |
| Total sugars (g) | 22.5 | 15 | 10 |
| Saturated fats (g) | 6 | 5 | 4 |
| **Liquids (per 100 ml)** |  |  |  |
| Energy (kcal) | 100 | 80 | 70 |
| Sodium (mg) | 100 | 100 | 100 |
| Total sugars (g) | 6 | 5 | 5 |
| Saturated fats (g) | 3 | 3 | 3 |
