# Supplementary material for: Decreases in purchases of energy, sodium, sugar, and saturated fat 3 years after implementation of the Chilean food labeling and marketing law: An interrupted time series analysis
Source: PLoS Med. 2024 Sep 27;21(9):e1004463. doi: 10.1371/journal.pmed.1004463 (PMC11432892; doi:10.1371/journal.pmed.1004463)
Supplement: S5 Table — Note: Direct matches are 1:1 barcode matches between the purchase and nutrition facts panel data. Products matched on other characteristics in the absence of a direct match are indirect matches. INTA, Institute of Nutrition and Food Technology; Mintel, Mintel Global New Products Database (Americas). (DOCX) [file pmed.1004463.s005.docx]

S5 Table. Nutrition Facts Panel source by linking period and match type.

|  | % SKUs | | | % Purchases | | |
| --- | --- | --- | --- | --- | --- | --- |
|  | Direct | Indirect | Total | Direct | Indirect | Total |
| Jan 2013-Jun 2016 (pre) |  |  |  |  |  |  |
| INTA 2015 | 14 | 34 | 48 | 39 | 15 | 54 |
| INTA 2016 | 14 | 28 | 42 | 24 | 15 | 39 |
| Mintel | 4 | 5 | 9 | 4 | 3 | 7 |
| Total | 32 | 68 | 100 | 67 | 33 | 100 |
| Jul 2016-Jun 2017 (phase 1) |  |  |  |  |  |  |
| INTA 2015 | 4 | 5 | 9 | 4 | 2 | 7 |
| INTA 2016 | 10 | 9 | 19 | 10 | 6 | 15 |
| INTA 2017 | 29 | 35 | 64 | 49 | 24 | 74 |
| Mintel | 5 | 2 | 8 | 2 | 2 | 4 |
| Total | 49 | 51 | 100 | 66 | 34 | 100 |
| Jul 2017-Jun 2018 (phase 1) |  |  |  |  |  |  |
| INTA 2015 | 2 | 1 | 3 | 1 | 0 | 2 |
| INTA 2016 | 4 | 2 | 5 | 1 | 0 | 1 |
| INTA 2017 | 5 | 3 | 8 | 4 | 1 | 6 |
| INTA 2018 | 44 | 32 | 76 | 73 | 15 | 88 |
| Mintel | 4 | 3 | 7 | 2 | 2 | 3 |
| Total | 60 | 40 | 100 | 82 | 18 | 100 |
| Jul 2018-Jun 2019 (phase 2) |  |  |  |  |  |  |
| INTA 2015 | 1 | 1 | 2 | 1 | 0 | 1 |
| INTA 2016 | 2 | 1 | 3 | 0 | 0 | 0 |
| INTA 2017 | 3 | 1 | 4 | 1 | 0 | 1 |
| INTA 2018 | 9 | 3 | 12 | 5 | 1 | 5 |
| INTA 2019 | 45 | 28 | 73 | 77 | 12 | 89 |
| Mintel | 4 | 3 | 7 | 2 | 1 | 3 |
| Total | 64 | 36 | 100 | 85 | 15 | 100 |

Note: direct matches are 1:1 barcode matches between the purchase and nutrition facts panel data. Products matched on other characteristics in the absence of a direct match are indirect matches. INTA= Institute of Nutrition and Food Technology. Mintel= Mintel Global New Products Database (Americas).
