# Supplementary material for: Decreases in purchases of energy, sodium, sugar, and saturated fat 3 years after implementation of the Chilean food labeling and marketing law: An interrupted time series analysis
Source: PLoS Med. 2024 Sep 27;21(9):e1004463. doi: 10.1371/journal.pmed.1004463 (PMC11432892; doi:10.1371/journal.pmed.1004463)
Supplement: S6 Table — Notes: Initial education level: education level in 2013 or enrollment year for new participants. CASEN: Chile Encuesta Nacional de Caracterización Socio-económica. e: extrapolated from the nearest 2 estimates assuming a linear change. CASEN totals do not necessarily sum to 100 due to missing values. (DOCX) [file pmed.1004463.s006.docx]

S6 Table. Changes in the distribution of households by household head’s current and initial education levels (% of households) in the weighted sample compared to national estimates for the urban population from CASEN.

|  | 2013 | 2014 | 2015 | 2016 | 2017 | 2018 | 2019 | 2020 |
| --- | --- | --- | --- | --- | --- | --- | --- | --- |
| Weighted sample, current education |  |  |  |  |  |  |  |  |
| < High school (HS) | 42 | 40 | 37 | 32 | 31 | 28 | 27 |  |
| = HS | 37 | 38 | 40 | 42 | 42 | 45 | 44 |  |
| > HS | 21 | 22 | 23 | 25 | 27 | 28 | 29 |  |
| Total | 100 | 100 | 100 | 100 | 100 | 100 | 100 |  |
| Weighted sample, initial education |  |  |  |  |  |  |  |  |
| < HS | 42 | 41 | 39 | 37 | 36 | 37 | 36 |  |
| = HS | 37 | 37 | 38 | 40 | 41 | 39 | 40 |  |
| > HS | 21 | 21 | 22 | 23 | 23 | 24 | 24 |  |
| Total | 100 | 100 | 100 | 100 | 100 | 100 | 100 |  |
| CASEN (urban) |  |  |  |  |  |  |  |  |
| < HS | 44 | *^e^*43 | 42 | *^e^*41 | 40 | *^e^*38 | *^e^*36 | 34 |
| = HS | 35 | *^e^*35 | 36 | *^e^*36 | 36 | *^e^*36 | *^e^*36 | 36 |
| > HS | 21 | *^e^*21 | 22 | *^e^*22 | 23 | *^e^*25 | *^e^*27 | 28 |
| Total | 100 | 100 | 100 | 100 | 99 | 99 | 99 | 98 |

Notes: Initial education level: education level in 2013 or enrollment year for new participants. CASEN: Encuesta de Caracterización Socioeconómica Nacional. *^e^*: extrapolated from the nearest two estimates assuming a linear change. CASEN totals do not necessarily sum to 100 due to missing values.
