# Supplementary material for: Decreases in purchases of energy, sodium, sugar, and saturated fat 3 years after implementation of the Chilean food labeling and marketing law: An interrupted time series analysis
Source: PLoS Med. 2024 Sep 27;21(9):e1004463. doi: 10.1371/journal.pmed.1004463 (PMC11432892; doi:10.1371/journal.pmed.1004463)
Supplement: S7 Table — Notes: NOC are sugar, saturated fat, sodium, and energy. As a result of the nutrition facts panel data linking protocol, the number of excess NOCs a product has may vary within a given period (multiple years of nutrition facts panel data used in the pre-policy and Phase 1 periods). (DOCX) [file pmed.1004463.s007.docx]

S7 Table. Raw percentages of products with zero, one, and two or more excess nutrients of concern (NOC) (Phase 3 limits) by period (July 2013-June 2019).

|  | Pre-policy | Phase 1 | Phase 2 |
| --- | --- | --- | --- |
| Food |  |  |  |
| 0 high-in NOC | 30.8% | 34.8% | 38.6% |
| 1 high-in NOC | 24.9% | 26.5% | 24.2% |
| 2 high-in NOCs | 44.2% | 38.7% | 37.2% |
| Beverages |  |  |  |
| 0 high-in NOC | 58.4% | 76.2% | 82.7% |
| 1 high-in NOC | 39.0% | 22.5% | 16.8% |
| 2 high-in excess NOCs | 2.5% | 1.2% | 0.5% |
| Total |  |  |  |
| 0 high-in NOC | 39.3% | 47.2% | 51.9% |
| 1 high-in NOC | 29.3% | 25.3% | 22.0% |
| 2 high-in NOCs | 31.4% | 27.5% | 26.1% |

Notes: NOC are sugar, saturated fat, sodium and energy. As a result of the nutrition facts panel data linking protocol, the number of excess NOCs a product has may vary within a given period (multiple years of nutrition facts panel data used in the pre-policy and phase 1 periods).
