# Supplementary material for: Decreases in purchases of energy, sodium, sugar, and saturated fat 3 years after implementation of the Chilean food labeling and marketing law: An interrupted time series analysis
Source: PLoS Med. 2024 Sep 27;21(9):e1004463. doi: 10.1371/journal.pmed.1004463 (PMC11432892; doi:10.1371/journal.pmed.1004463)
Supplement: S8 Table — (DOCX) [file pmed.1004463.s008.docx]

S8 Table. Unadjusted weighted means (Standard Deviation) in food and beverage purchases by July-June period.

|  | **2013-2014** | **2014-2015** | **2015-2016** | **2016-2017** | **2017-2018** | **2018-2019** |
| --- | --- | --- | --- | --- | --- | --- |
| **Total** |  |  |  |  |  |  |
| Energy (calories/capita/day) |  |  |  |  |  |  |
| High-in | 218.4 | 228.3 | 233.8 | 191.7 | 198.5 | 177.9 |
|  | (151.0) | (161.7) | (166.3) | (139.3) | (144.3) | (132.7) |
| Not high-in | 214.5 | 216.2 | 223.4 | 221.4 | 217.8 | 232.0 |
|  | (166.6) | (181.5) | (186.1) | (178.9) | (184.8) | (191.1) |
| Total | 432.9 | 444.5 | 457.3 | 413.1 | 416.3 | 409.9 |
|  | (277.2) | (303.7) | (311.5) | (278.7) | (289.3) | (288.1) |
| High-in share | 52.0% | 53.2% | 52.9% | 48.1% | 50.0% | 45.7% |
|  | (18.1%) | (18.0%) | (18.0%) | (18.3%) | (18.7%) | (18.6%) |
| Saturated fat (calories/capita/day) |  |  |  |  |  |  |
| High-in | 35.1 | 37.1 | 39.6 | 35.1 | 37.1 | 34.5 |
|  | (28.2) | (31.4) | (33.9) | (31.2) | (32.9) | (32.0) |
| Not high-in | 10.6 | 10.7 | 11.3 | 11.8 | 11.2 | 13.2 |
|  | (12.9) | (13.5) | (14.4) | (14.3) | (13.6) | (15.7) |
| Total | 45.7 | 47.8 | 50.9 | 47.0 | 48.2 | 47.7 |
|  | (35.2) | (38.9) | (41.8) | (38.9) | (40.3) | (41.3) |
| High-in share | 77.3% | 78.1% | 78.2% | 74.0% | 76.0% | 71.7% |
|  | (18.8%) | (18.5%) | (18.4%) | (19.9%) | (19.2%) | (20.4%) |
| Sodium (sodium/capita/day) |  |  |  |  |  |  |
| High-in | 412.7 | 422.7 | 429.1 | 357.6 | 349.1 | 314.1 |
|  | (353.3) | (379.6) | (378.8) | (324.9) | (319.3) | (306.9) |
| Not high-in | 160.5 | 159.0 | 161.5 | 157.7 | 146.7 | 159.4 |
|  | (190.9) | (204.4) | (204.2) | (177.5) | (176.7) | (179.4) |
| Total | 573.2 | 581.7 | 590.6 | 515.3 | 495.8 | 473.5 |
|  | (464.2) | (500.6) | (496.9) | (428.8) | (421.1) | (412.9) |
| High-in share | 72.4% | 73.3% | 72.9% | 68.7% | 70.0% | 65.2% |
|  | (19.2%) | (18.9%) | (19.1%) | (19.6%) | (19.5%) | (20.3%) |
| Sugars (calories/capita/day) |  |  |  |  |  |  |
| High-in | 85.5 | 89.5 | 87.5 | 64.9 | 66.1 | 54.5 |
|  | (71.6) | (77.2) | (74.2) | (59.5) | (59.0) | (51.8) |
| Not high-in | 24.0 | 23.4 | 24.1 | 28.2 | 26.4 | 32.1 |
|  | (22.5) | (22.6) | (23.1) | (24.6) | (23.3) | (27.6) |
| Total | 109.4 | 112.9 | 111.6 | 93.1 | 92.5 | 86.6 |
|  | (81.7) | (86.9) | (85.0) | (71.5) | (70.4) | (66.3) |
| High-in share | 75.9% | 77.1% | 76.0% | 66.6% | 68.4% | 59.4% |
|  | (16.9%) | (16.6%) | (17.0%) | (19.4%) | (19.0%) | (21.2%) |
| **Foods** |  |  |  |  |  |  |
| Energy (calories/capita/day) |  |  |  |  |  |  |
| High-in | 161.1 | 167.3 | 175.3 | 154.1 | 160.4 | 149.1 |
|  | (118.8) | (127.5) | (134.2) | (119.5) | (126.1) | (120.1) |
| Not high-in | 171.5 | 173.7 | 180.3 | 174.7 | 175.0 | 184.5 |
|  | (144.2) | (158.5) | (161.4) | (154.3) | (162.2) | (165.3) |
| Total | 332.7 | 341.1 | 355.6 | 328.8 | 335.4 | 333.6 |
|  | (226.2) | (249.2) | (257.5) | (235.3) | (249.2) | (249.6) |
| High-in share | 50.7% | 51.6% | 51.7% | 49.3% | 51.0% | 47.7% |
|  | (20.7%) | (20.8%) | (20.7%) | (21.0%) | (21.5%) | (21.2%) |
| Saturated fat (calories/capita/day) |  |  |  |  |  |  |
| High-in | 34.2 | 36.1 | 38.5 | 35.0 | 36.2 | 34.4 |
|  | (27.8) | (30.9) | (33.4) | (31.1) | (32.4) | (31.9) |
| Not high-in | 3.2 | 3.3 | 3.6 | 4.3 | 4.1 | 5.6 |
|  | (5.9) | (6.1) | (6.5) | (6.8) | (7.2) | (9.3) |
| Total | 37.4 | 39.4 | 42.2 | 39.3 | 40.3 | 40.0 |
|  | (30.2) | (33.4) | (36.0) | (33.9) | (35.2) | (36.3) |
| High-in share | 90.7% | 90.7% | 90.5% | 87.2% | 88.9% | 84.9% |
|  | (12.4%) | (12.8%) | (12.8%) | (14.9%) | (14.3%) | (16.5%) |
| Sodium (sodium/capita/day) |  |  |  |  |  |  |
| High-in | 393.2 | 402.3 | 409.4 | 347.1 | 337.1 | 307.3 |
|  | (346.6) | (372.5) | (371.8) | (321.7) | (315.6) | (305.5) |
| Not high-in | 117.7 | 116.8 | 118.4 | 113.0 | 105.8 | 115.0 |
|  | (173.8) | (187.8) | (186.3) | (160.1) | (160.9) | (161.5) |
| Total | 510.9 | 519.1 | 527.8 | 460.1 | 442.9 | 422.3 |
|  | (436.8) | (471.9) | (466.8) | (404.6) | (398.6) | (391.5) |
| High-in share | 78.5% | 79.3% | 78.8% | 76.0% | 77.2% | 73.0% |
|  | (20.6%) | (20.4%) | (20.5%) | (20.8%) | (20.6%) | (21.6%) |
| Sugars (calories/capita/day) |  |  |  |  |  |  |
| High-in | 32.8 | 33.4 | 33.8 | 28.8 | 31.1 | 26.5 |
|  | (27.5) | (28.2) | (29.4) | (26.3) | (28.9) | (25.5) |
| Not high-in | 4.6 | 4.6 | 5.6 | 7.1 | 7.5 | 10.5 |
|  | (5.2) | (5.4) | (6.2) | (7.1) | (7.6) | (11.1) |
| Total | 37.4 | 38.0 | 39.4 | 35.9 | 38.6 | 37.0 |
|  | (29.9) | (30.7) | (32.3) | (29.6) | (32.6) | (31.5) |
| High-in share | 84.8% | 85.0% | 82.3% | 76.1% | 76.4% | 67.8% |
|  | (15.0%) | (15.3%) | (16.8%) | (19.4%) | (19.4%) | (21.7%) |
| **Beverages** |  |  |  |  |  |  |
| Energy (calories/capita/day) |  |  |  |  |  |  |
| High-in | 57.3 | 61.0 | 58.5 | 37.6 | 38.2 | 28.8 |
|  | (62.3) | (69.1) | (64.7) | (50.4) | (51.6) | (43.5) |
| Not high-in | 42.9 | 42.5 | 43.2 | 46.8 | 42.8 | 47.5 |
|  | (46.3) | (47.8) | (50.0) | (50.4) | (46.6) | (50.0) |
| Total | 100.2 | 103.4 | 101.7 | 84.3 | 80.9 | 76.3 |
|  | (84.3) | (90.9) | (89.6) | (76.4) | (74.1) | (69.9) |
| High-in share | 55.4% | 57.3% | 56.4% | 42.1% | 43.4% | 34.0% |
|  | (28.2%) | (28.1%) | (28.1%) | (29.4%) | (30.4%) | (30.6%) |
| Saturated fat (calories/capita/day) |  |  |  |  |  |  |
| High-in | 1.0 | 1.0 | 1.1 | 0.2 | 0.8 | 0.1 |
|  | (1.9) | (2.1) | (2.2) | (0.7) | (5.2) | (1.1) |
| Not high-in | 7.4 | 7.3 | 7.6 | 7.5 | 7.1 | 7.6 |
|  | (10.5) | (11.0) | (11.8) | (11.4) | (10.3) | (11.1) |
| Total | 8.3 | 8.4 | 8.7 | 7.7 | 7.9 | 7.7 |
|  | (10.9) | (11.6) | (12.3) | (11.5) | (11.8) | (11.2) |
| High-in share | 24.4% | 25.6% | 24.8% | 4.9% | 5.5% | 2.1% |
|  | (34.4%) | (35.2%) | (34.5%) | (16.9%) | (19.4%) | (11.3%) |
| Sodium (sodium/capita/day) |  |  |  |  |  |  |
| High-in | 19.5 | 20.4 | 19.8 | 10.5 | 12.0 | 6.8 |
|  | (21.7) | (23.5) | (22.4) | (13.9) | (19.5) | (11.2) |
| Not high-in | 42.8 | 42.2 | 43.1 | 44.7 | 40.9 | 44.4 |
|  | (46.9) | (47.3) | (49.4) | (48.0) | (44.3) | (47.0) |
| Total | 62.3 | 62.6 | 62.8 | 55.1 | 52.9 | 51.2 |
|  | (55.7) | (56.9) | (58.6) | (51.9) | (50.5) | (49.2) |
| High-in share | 37.4% | 38.9% | 37.9% | 24.4% | 26.3% | 17.7% |
|  | (28.0%) | (28.2%) | (27.7%) | (24.4%) | (25.8%) | (22.9%) |
| Sugars (calories/capita/day) |  |  |  |  |  |  |
| High-in | 52.7 | 56.1 | 53.7 | 36.1 | 35.1 | 27.9 |
|  | (59.1) | (65.5) | (61.0) | (49.2) | (47.5) | (42.7) |
| Not high-in | 19.3 | 18.9 | 18.5 | 21.1 | 18.9 | 21.6 |
|  | (20.3) | (20.3) | (20.3) | (21.4) | (19.5) | (21.6) |
| Total | 72.0 | 74.9 | 72.2 | 57.2 | 53.9 | 49.6 |
|  | (65.7) | (71.4) | (67.6) | (56.4) | (53.6) | (50.0) |
| High-in share | 66.4% | 68.2% | 67.8% | 53.4% | 54.4% | 43.6% |
|  | (27.4%) | (27.0%) | (27.1%) | (31.2%) | (32.0%) | (33.8%) |
