# Supplementary material for: Decreases in purchases of energy, sodium, sugar, and saturated fat 3 years after implementation of the Chilean food labeling and marketing law: An interrupted time series analysis
Source: PLoS Med. 2024 Sep 27;21(9):e1004463. doi: 10.1371/journal.pmed.1004463 (PMC11432892; doi:10.1371/journal.pmed.1004463)
Supplement: S9 Table — (DOCX) [file pmed.1004463.s009.docx]

S9 Table. Unadjusted weighted mean (Standard Deviation) nutrient content of food and beverage purchases by policy period and socioeconomic status (SES).

|  | Pre-policy | | Phase 1 | | Phase 2 | |
| --- | --- | --- | --- | --- | --- | --- |
|  | High SES | Low SES | High SES | Low SES | High SES | Low SES |
| **Total** |  |  |  |  |  |  |
| Energy |  |  |  |  |  |  |
| High-in | 263.7 | 215.2 | 216.5 | 188.3 | 190.5 | 173.9 |
|  | (179.5) | (151.4) | (155.3) | (136.6) | (143.7) | (128.8) |
| Not high-in | 231.4 | 213.8 | 228.4 | 216.8 | 241.0 | 229.2 |
|  | (186.7) | (175.5) | (180.5) | (182.2) | (194.6) | (189.9) |
| Total | 495.2 | 429.0 | 445.0 | 405.1 | 431.4 | 403.1 |
|  | (326.7) | (286.6) | (294.0) | (280.1) | (301.8) | (283.3) |
| High-in share | 54.7% | 52.1% | 50.0% | 48.8% | 45.8% | 45.6% |
|  | (16.5%) | (18.5%) | (17.5%) | (18.8%) | (18.0%) | (18.8%) |
| Saturated fat |  |  |  |  |  |  |
| High-in | 46.4 | 34.4 | 44.4 | 33.5 | 42.8 | 31.9 |
|  | (37.4) | (28.5) | (38.7) | (29.2) | (40.1) | (28.5) |
| Not high-in | 13.4 | 10.0 | 13.8 | 10.8 | 16.2 | 12.2 |
|  | (15.7) | (12.8) | (16.0) | (13.2) | (19.6) | (14.2) |
| Total | 59.8 | 44.4 | 58.2 | 44.2 | 59.0 | 44.2 |
|  | (45.9) | (35.5) | (46.9) | (36.3) | (51.2) | (36.9) |
| High-in share | 77.6% | 78.0% | 75.3% | 74.9% | 71.1% | 71.9% |
|  | (17.9%) | (18.8%) | (18.8%) | (19.8%) | (20.3%) | (20.4%) |
| Sodium |  |  |  |  |  |  |
| High-in | 464.3 | 408.0 | 375.2 | 346.4 | 310.3 | 315.3 |
|  | (407.6) | (357.4) | (336.4) | (317.1) | (301.3) | (308.6) |
| Not high-in | 195.5 | 149.1 | 178.9 | 143.6 | 192.3 | 149.1 |
|  | (227.0) | (189.2) | (197.1) | (169.5) | (210.0) | (167.3) |
| Total | 659.8 | 557.0 | 554.2 | 490.0 | 502.6 | 464.3 |
|  | (547.5) | (464.2) | (455.6) | (413.7) | (439.1) | (403.9) |
| High-in share | 71.1% | 73.4% | 67.3% | 70.0% | 60.9% | 66.6% |
|  | (18.3%) | (19.3%) | (19.0%) | (19.7%) | (20.2%) | (20.2%) |
| Sugars |  |  |  |  |  |  |
| High-in | 91.9 | 86.1 | 63.7 | 66.1 | 49.5 | 56.0 |
|  | (75.4) | (74.0) | (59.6) | (59.1) | (48.3) | (52.8) |
| Not high-in | 28.4 | 22.4 | 31.4 | 25.9 | 36.4 | 30.8 |
|  | (26.3) | (21.3) | (27.1) | (22.7) | (32.0) | (25.9) |
| Total | 120.2 | 108.5 | 95.2 | 92.0 | 85.9 | 86.8 |
|  | (89.0) | (82.9) | (73.4) | (70.1) | (66.9) | (66.1) |
| High-in share | 74.6% | 76.9% | 63.9% | 68.6% | 54.8% | 60.8% |
|  | (16.9%) | (16.8%) | (19.7%) | (18.9%) | (21.5%) | (20.9%) |
| **Foods** |  |  |  |  |  |  |
| Energy |  |  |  |  |  |  |
| High-in | 206.2 | 155.8 | 184.8 | 148.5 | 169.3 | 142.7 |
|  | (146.1) | (118.0) | (136.5) | (116.8) | (133.6) | (114.7) |
| Not high-in | 179.9 | 173.7 | 175.9 | 174.5 | 186.9 | 183.8 |
|  | (159.3) | (153.6) | (153.2) | (159.9) | (163.3) | (165.9) |
| Total | 386.1 | 329.5 | 360.6 | 322.9 | 356.3 | 326.5 |
|  | (267.5) | (235.7) | (248.3) | (239.7) | (260.6) | (245.5) |
| High-in share | 55.4% | 50.1% | 53.2% | 49.1% | 49.6% | 47.1% |
|  | (18.8%) | (21.2%) | (19.7%) | (21.6%) | (20.2%) | (21.5%) |
| Saturated fat |  |  |  |  |  |  |
| High-in | 45.2 | 33.5 | 44.0 | 32.9 | 42.7 | 31.8 |
|  | (37.0) | (28.1) | (38.5) | (28.8) | (40.0) | (28.4) |
| Not high-in | 5.1 | 2.9 | 6.0 | 3.6 | 8.6 | 4.7 |
|  | (8.3) | (5.2) | (9.6) | (5.9) | (13.8) | (7.0) |
| Total | 50.3 | 36.3 | 50.0 | 36.6 | 51.3 | 36.5 |
|  | (40.2) | (30.1) | (42.1) | (31.1) | (46.3) | (31.7) |
| High-in share | 89.1% | 91.1% | 86.6% | 88.5% | 81.7% | 85.9% |
|  | (13.1%) | (12.5%) | (15.0%) | (14.5%) | (17.9%) | (15.9%) |
| Sodium |  |  |  |  |  |  |
| High-in | 442.9 | 388.6 | 365.0 | 334.8 | 305.2 | 307.9 |
|  | (399.1) | (351.0) | (332.3) | (313.9) | (300.1) | (307.1) |
| Not high-in | 137.8 | 111.2 | 123.5 | 104.9 | 135.9 | 108.5 |
|  | (204.4) | (174.9) | (175.8) | (155.1) | (185.1) | (152.8) |
| Total | 580.7 | 499.8 | 488.4 | 439.7 | 441.1 | 416.4 |
|  | (512.6) | (438.6) | (426.5) | (392.8) | (410.8) | (385.1) |
| High-in share | 78.2% | 79.0% | 75.9% | 76.9% | 69.7% | 74.1% |
|  | (19.7%) | (20.7%) | (20.2%) | (20.9%) | (21.5%) | (21.4%) |
| Sugars |  |  |  |  |  |  |
| High-in | 40.2 | 31.1 | 34.2 | 28.6 | 29.2 | 25.7 |
|  | (33.2) | (26.3) | (30.0) | (26.7) | (27.3) | (24.9) |
| Not high-in | 6.0 | 4.6 | 7.9 | 7.1 | 11.7 | 10.1 |
|  | (6.8) | (5.2) | (7.9) | (7.2) | (13.1) | (10.4) |
| Total | 46.2 | 35.8 | 42.1 | 35.7 | 40.8 | 35.8 |
|  | (36.3) | (28.6) | (33.4) | (30.3) | (33.8) | (30.6) |
| High-in share | 84.5% | 83.9% | 77.4% | 75.9% | 68.3% | 67.7% |
|  | (14.6%) | (16.2%) | (18.1%) | (19.8%) | (21.3%) | (21.8%) |
| **Beverages** |  |  |  |  |  |  |
| Energy |  |  |  |  |  |  |
| High-in | 57.5 | 59.4 | 31.8 | 39.8 | 21.1 | 31.2 |
|  | (62.2) | (66.4) | (48.4) | (51.7) | (37.6) | (45.0) |
| Not high-in | 51.6 | 40.1 | 52.5 | 42.3 | 54.0 | 45.4 |
|  | (54.3) | (45.5) | (53.8) | (46.5) | (56.4) | (47.7) |
| Total | 109.1 | 99.5 | 84.3 | 82.1 | 75.1 | 76.6 |
|  | (92.3) | (86.9) | (77.7) | (74.5) | (71.8) | (69.3) |
| High-in share | 51.8% | 57.9% | 34.4% | 45.4% | 24.6% | 36.9% |
|  | (28.4%) | (27.9%) | (29.5%) | (29.6%) | (28.6%) | (30.6%) |
| Saturated fat |  |  |  |  |  |  |
| High-in | 1.3 | 0.9 | 0.4 | 0.5 | 0.1 | 0.1 |
|  | (2.6) | (1.9) | (3.1) | (3.9) | (1.0) | (1.1) |
| Not high-in | 8.3 | 7.2 | 7.8 | 7.1 | 7.6 | 7.5 |
|  | (12.0) | (10.8) | (11.4) | (10.7) | (11.7) | (10.9) |
| Total | 9.6 | 8.1 | 8.2 | 7.7 | 7.7 | 7.7 |
|  | (12.5) | (11.3) | (11.9) | (11.5) | (11.7) | (11.0) |
| High-in share | 24.3% | 25.1% | 4.9% | 5.3% | 1.7% | 2.2% |
|  | (34.1%) | (34.9%) | (17.7%) | (18.4%) | (10.0%) | (11.7%) |
| Sodium |  |  |  |  |  |  |
| High-in | 21.4 | 19.4 | 10.3 | 11.5 | 5.1 | 7.3 |
|  | (24.8) | (21.7) | (17.8) | (16.7) | (10.3) | (11.4) |
| Not high-in | 57.8 | 37.9 | 55.4 | 38.8 | 56.4 | 40.6 |
|  | (59.8) | (42.3) | (55.5) | (42.0) | (58.7) | (42.0) |
| Total | 79.1 | 57.3 | 65.7 | 50.3 | 61.6 | 47.9 |
|  | (69.5) | (51.4) | (60.3) | (47.3) | (60.2) | (44.7) |
| High-in share | 32.6% | 39.8% | 19.0% | 27.4% | 11.5% | 19.7% |
|  | (26.4%) | (28.2%) | (22.4%) | (25.6%) | (19.0%) | (23.7%) |
| Sugars |  |  |  |  |  |  |
| High-in | 51.7 | 55.0 | 29.6 | 37.5 | 20.4 | 30.3 |
|  | (57.7) | (63.3) | (45.6) | (49.0) | (36.3) | (44.2) |
| Not high-in | 22.3 | 17.8 | 23.5 | 18.9 | 24.8 | 20.7 |
|  | (23.4) | (19.1) | (23.6) | (19.3) | (25.1) | (20.3) |
| Total | 74.0 | 72.7 | 53.1 | 56.3 | 45.1 | 51.0 |
|  | (67.0) | (68.7) | (54.3) | (55.2) | (46.7) | (50.9) |
| High-in share | 63.5% | 68.8% | 44.7% | 56.8% | 32.5% | 47.2% |
|  | (28.3%) | (26.7%) | (32.6%) | (30.7%) | (33.2%) | (33.3%) |
