# Supplementary material for: Decreases in purchases of energy, sodium, sugar, and saturated fat 3 years after implementation of the Chilean food labeling and marketing law: An interrupted time series analysis
Source: PLoS Med. 2024 Sep 27;21(9):e1004463. doi: 10.1371/journal.pmed.1004463 (PMC11432892; doi:10.1371/journal.pmed.1004463)
Supplement: S10 Table — Notes: *** p < .01, ** p < .05, * p < .1. Standard errors in parentheses. The July 2018 (Phase 2) estimates are relative to the Phase 1 trend; for example, a nonzero July 2018 slope change indicates a change in the slope of the trend from phase 1. Adjusted for seasonality (month dummies), household composition (number of household members by age and sex group), SES (4 categories), head of household education level (less than high school, high school, more than high school), region-quarter unemployment rate, number of public holidays in the month, October 2014 beverage tax changes (pre-post dummy), and unobserved time-invariant household characteristics. N = 138,367 household-month obs. (2,842 households). (DOCX) [file pmed.1004463.s010.docx]

S10 Table. Impact model parameter estimates (percentage changes).

|  | Energy | | Saturated fat | | Sodium | | Sugars | |
| --- | --- | --- | --- | --- | --- | --- | --- | --- |
|  | Intercept  change | Slope  change | Intercept  change | Slope  change | Intercept  change | Slope  change | Intercept  change | Slope  change |
| **Total** |  |  |  |  |  |  |  |  |
| July 2016 |  |  |  |  |  |  |  |  |
| High-in | -20.5*** | 0.4*** | -14.5*** | 0.4*** | -16.1*** | 0.1 | -28.5*** | 0.2** |
|  | (0.7) | (0.1) | (0.9) | (0.1) | (1.0) | (0.1) | (0.8) | (0.1) |
| Not high-in | -0.5 | 0.0 | 4.4** | -0.4*** | -0.2 | -0.2 | 19.4*** | -0.4*** |
|  | (1.0) | (0.1) | (1.7) | (0.1) | (1.7) | (0.1) | (1.6) | (0.1) |
| Total | -10.8*** | 0.2*** | -10.4*** | 0.2** | -11.7*** | 0.0 | -18.1*** | 0.1 |
|  | (0.7) | (0.1) | (0.8) | (0.1) | (0.9) | (0.1) | (0.8) | (0.1) |
| July 2018 |  |  |  |  |  |  |  |  |
| High-in | -5.9*** | -1.4*** | -3.5** | -1.5*** | -2.8* | -1.3*** | -11.3*** | -1.2*** |
|  | (1.1) | (0.1) | (1.4) | (0.2) | (1.6) | (0.2) | (1.4) | (0.2) |
| Not high-in | 14.0*** | -1.3*** | 24.7*** | -0.5** | 19.8*** | -1.0*** | 36.0*** | -1.1*** |
|  | (1.5) | (0.2) | (2.7) | (0.2) | (2.4) | (0.3) | (2.2) | (0.2) |
| Total | 4.3*** | -1.3*** | 2.8** | -1.3*** | 4.0*** | -1.2*** | 1.8 | -1.1*** |
|  | (1.1) | (0.1) | (1.3) | (0.2) | (1.5) | (0.2) | (1.2) | (0.2) |
| **Foods** |  |  |  |  |  |  |  |  |
| July 2016 |  |  |  |  |  |  |  |  |
| High-in | -14.4*** | 0.4*** | -12.3*** | 0.3*** | -14.4*** | 0.1 | -17.2*** | 0.8*** |
|  | (0.8) | (0.1) | (0.9) | (0.1) | (1.0) | (0.1) | (1.0) | (0.1) |
| Not high-in | -3.4*** | 0.2** | 15.3*** | -0.2 | -2.2 | -0.1 | 13.8*** | -0.2* |
|  | (1.0) | (0.1) | (2.9) | (0.2) | (2.2) | (0.2) | (1.9) | (0.1) |
| Total | -8.9*** | 0.3*** | -9.8*** | 0.2** | -11.7*** | 0.0 | -12.5*** | 0.7*** |
|  | (0.7) | (0.1) | (0.9) | (0.1) | (0.9) | (0.1) | (1.0) | (0.1) |
| July 2018 |  |  |  |  |  |  |  |  |
| High-in | -3.0** | -1.6*** | -1.2 | -1.5*** | -1.3 | -1.3*** | -9.8*** | -2.0*** |
|  | (1.2) | (0.2) | (1.4) | (0.2) | (1.6) | (0.2) | (1.5) | (0.2) |
| Not high-in | 11.2*** | -1.4*** | 38.2*** | -0.3 | 19.8*** | -1.2*** | 35.6*** | -1.3*** |
|  | (1.6) | (0.2) | (4.1) | (0.4) | (3.1) | (0.3) | (3.0) | (0.2) |
| Total | 4.2*** | -1.5*** | 2.8** | -1.3*** | 3.9** | -1.3*** | -1.1 | -1.8*** |
|  | (1.2) | (0.1) | (1.4) | (0.2) | (1.6) | (0.2) | (1.4) | (0.2) |
| **Beverages** |  |  |  |  |  |  |  |  |
| July 2016 |  |  |  |  |  |  |  |  |
| High-in | -39.3*** | 0.0 |  |  |  |  | -35.7*** | -0.3** |
|  | (1.1) | (0.1) |  |  |  |  | (1.1) | (0.1) |
| Not high-in | 11.5*** | -0.6*** |  |  |  |  | 21.0*** | -0.6*** |
|  | (1.7) | (0.1) |  |  |  |  | (1.8) | (0.1) |
| Total | -17.3*** | -0.3*** |  |  |  |  | -21.1*** | -0.4*** |
|  | (0.9) | (0.1) |  |  |  |  | (1.0) | (0.1) |
| July 2018 |  |  |  |  |  |  |  |  |
| High-in | -19.9*** | -0.7** |  |  |  |  | -13.4*** | -0.4 |
|  | (2.0) | (0.3) |  |  |  |  | (2.0) | (0.3) |
| Not high-in | 25.6*** | -0.8*** |  |  |  |  | 34.5*** | -1.0*** |
|  | (2.4) | (0.2) |  |  |  |  | (2.5) | (0.2) |
| Total | 4.2*** | -0.7*** |  |  |  |  | 3.3** | -0.6*** |
|  | (1.5) | (0.2) |  |  |  |  | (1.5) | (0.2) |

Notes: *** p < .01, ** p < .05, * p < .1. Standard errors in parentheses. The July 2018 (phase 2) estimates are relative to the phase 1 trend; for example, a non-zero July 2018 slope change indicates a change in the slope of the trend from phase 1. Adjusted for seasonality (month dummies), household composition (number of household members by age and sex group), SES (four categories), head of household education level (less than high school, high school, more than high school), region-quarter unemployment rate, number of public holidays in the month, October 2014 beverage tax changes (pre-post dummy), and unobserved time-invariant household characteristics. N = 138,367 household-month obs. (2,842 households).
