# Supplementary material for: Decreases in purchases of energy, sodium, sugar, and saturated fat 3 years after implementation of the Chilean food labeling and marketing law: An interrupted time series analysis
Source: PLoS Med. 2024 Sep 27;21(9):e1004463. doi: 10.1371/journal.pmed.1004463 (PMC11432892; doi:10.1371/journal.pmed.1004463)
Supplement: S11 Table — Notes: *** p < .01, ** p < .05, * p < .1. Standard errors in parentheses. The AICs and BICs are divided by 100 and rounded to the nearest integer to facilitate comparisons (omitted in (3)-(5) because each uses a different estimation dataset). All estimates are CRE Poisson and adjusted for seasonality (month dummies), household composition (number of household members by age and sex group), SES (4 categories), head of household education level (less than high school, high school, more than high school), region-quarter unemployment rate, number of public holidays in the month, October 2014 beverage tax changes (pre-post dummy), and unobserved time-invariant household characteristics (unless otherwise noted). (DOCX) [file pmed.1004463.s011.docx]

S11 Table. Sensitivity results (average percentage changes in total energy).

|  | (1) | (2) | (3) | (4) | (5) | (6) | (7) |
| --- | --- | --- | --- | --- | --- | --- | --- |
| Label rollout period (April-June 2016) | No | Yes | No | No | No | No | No |
| Baseline period (start date) | Jul 2013  (36 mo) | Jul 2013  (36 mo) | Jan 2014  (30 mo) | Jul 2014  (24 mo) | Jan 2015  (18 mo) | Jul 2013  (36 mo) | Jul 2013  (36 mo) |
| Adjusted for *current* household head education | Yes | Yes | Yes | Yes | Yes | No | No |
| Adjusted for *initial* household head education | No | No | No | No | No | No | Yes |
| *N* household-months | 138,367 | 138,367 | 126,731 | 115,057 | 103,276 | 138,367 | 138,367 |
| *N* households | 2,842 | 2,842 | 2,763 | 2,665 | 2,573 | 2,842 | 2,842 |
| **Total** |  |  |  |  |  |  |  |
| AIC | 214,070 | 214,045 |  |  |  | 214,389 | 214,225 |
| BIC | 214,077 | 214,051 |  |  |  | 214,394 | 214,231 |
| Phase 1 | -8.8*** | -10.3*** | -7.5*** | -7.4*** | -3.8*** | -8.8*** | -8.8*** |
|  | (0.9) | (1.0) | (1.0) | (1.0) | (1.2) | (0.9) | (0.9) |
| Phase 2 | -8.3*** | -10.8*** | -5.9*** | -5.6*** | 1.6 | -8.3*** | -8.3*** |
|  | (1.7) | (1.8) | (1.9) | (2.0) | (2.5) | (1.7) | (1.7) |
| **Foods** |  |  |  |  |  |  |  |
| AIC | 190,281 | 190,269 |  |  |  | 190,599 | 190,456 |
| BIC | 190,288 | 190,275 |  |  |  | 190,605 | 190,462 |
| Phase 1 | -5.5*** | -6.4*** | -5.1*** | -5.5*** | -1.8 | -5.5*** | -5.5*** |
|  | (1.0) | (1.1) | (1.1) | (1.1) | (1.4) | (1.0) | (1.0) |
| Phase 2 | -3.8** | -5.2** | -2.9 | -3.7* | 3.9 | -3.8** | -3.8** |
|  | (1.9) | (2.0) | (2.1) | (2.2) | (2.8) | (1.9) | (1.9) |
| **Beverages** |  |  |  |  |  |  |  |
| AIC | 85,191 | 85,171 |  |  |  | 85,218 | 85,178 |
| BIC | 85,197 | 85,177 |  |  |  | 85,224 | 85,184 |
| Phase 1 | -20.3*** | -23.6*** | -16.5*** | -14.6*** | -11.5*** | -20.3*** | -20.3*** |
|  | (1.2) | (1.3) | (1.3) | (1.4) | (1.6) | (1.2) | (1.2) |
| Phase 2 | -24.5*** | -29.5*** | -17.7*** | -14.4*** | -8.1** | -24.5*** | -24.4*** |
|  | (2.0) | (2.1) | (2.4) | (2.6) | (3.2) | (2.0) | (2.0) |

Notes: *** p < .01, ** p < .05, * p < .1. Standard errors in parentheses. The Akaike Information Criterions (AICs) and Bayesian Information Criterions (BICs) are divided by 100 and rounded to the nearest integer to facilitate comparisons (omitted in (3)-(5) because each uses a different estimation dataset). All estimates are correlated random-effects (CRE) Poisson and adjusted for seasonality (month dummies), household composition (number of household members by age and sex group), SES (four categories), current head of household education level (less than high school, high school, more than high school), region-quarter unemployment rate, number of public holidays in the month, October 2014 beverage tax changes (pre-post dummy), and unobserved time-invariant household characteristics (unless otherwise noted). Initial household head education: household head education in 2013 or at enrollment (excluded from Mundlak’s device).
