# Supplementary material for: Decreases in purchases of energy, sodium, sugar, and saturated fat 3 years after implementation of the Chilean food labeling and marketing law: An interrupted time series analysis
Source: PLoS Med. 2024 Sep 27;21(9):e1004463. doi: 10.1371/journal.pmed.1004463 (PMC11432892; doi:10.1371/journal.pmed.1004463)
Supplement: S12 Table — Note: Standard errors in parentheses. Adjusted for seasonality (month dummies), household composition (number of household members by age and sex group), SES (4 categories), head of household education level (less than high school, high school, more than high school), region-quarter unemployment rate, number of public holidays in the month, October 2014 beverage tax changes (pre-post dummy), and unobserved time-invariant household characteristics. N = 138,367 household-month obs. (2,842 households). (DOCX) [file pmed.1004463.s012.docx]

S12 Table. One-part vs. two-part model estimates for total energy by food and beverage sub-group (average difference with the counterfactual).

|  | Phase 1 | | Phase 2 | |
| --- | --- | --- | --- | --- |
|  | One-part | Two-part | One-part | Two-part |
| Breakfast cereals | -1.9 | -1.8 | -2.0 | -1.9 |
|  | (0.5) | (0.5) | (0.9) | (0.8) |
| Grain-based Desserts | -2.8 | -2.7 | -2.2 | -2.1 |
|  | (0.6) | (0.6) | (1.2) | (1.2) |
| Sweets and Non-grain-based Desserts | -1.6 | -1.5 | -0.9 | -0.9 |
|  | (0.5) | (0.5) | (0.9) | (0.9) |
| Meat, Poultry and Meat Substitutes | -2.1 | -2.0 | -2.4 | -2.1 |
|  | (0.6) | (0.6) | (1.1) | (1.1) |
| Dairy Products and Dairy Substitutes | -0.8 | -0.8 | -1.6 | -1.6 |
|  | (0.5) | (0.5) | (1.0) | (1.0) |
| Condiments and Sauces | -0.7 | -0.6 | 1.3 | 1.5 |
|  | (0.5) | (0.5) | (0.9) | (0.9) |
| Oils and Fats | -3.7 | -3.7 | -4.5 | -4.4 |
|  | (0.8) | (0.8) | (1.5) | (1.4) |
| Sodas | -8.1 | -8.1 | -9.4 | -9.2 |
|  | (0.9) | (0.9) | (1.5) | (1.5) |
| Industrialized, Fruit and Vegetable Juice (FVJ) | -6.5 | -6.4 | -9.2 | -8.7 |
|  | (0.5) | (0.4) | (0.9) | (0.8) |
| Dairy-based Beverages and Dairy Substitutes | -7.7 | -7.7 | -9.3 | -9.4 |
|  | (1.0) | (1.0) | (1.8) | (1.8) |

Note: Standard errors in parentheses. Adjusted for seasonality (month dummies), household composition (number of household members by age and sex group), SES (four categories), head of household education level (less than high school, high school, more than high school), region-quarter unemployment rate, number of public holidays in the month, October 2014 beverage tax changes (pre-post dummy), and unobserved time-invariant household characteristics. N = 138,367 household-month obs. (2,842 households).
