# Supplementary material for: Decreases in purchases of energy, sodium, sugar, and saturated fat 3 years after implementation of the Chilean food labeling and marketing law: An interrupted time series analysis
Source: PLoS Med. 2024 Sep 27;21(9):e1004463. doi: 10.1371/journal.pmed.1004463 (PMC11432892; doi:10.1371/journal.pmed.1004463)
Supplement: S1 Fig — (DOCX) [file pmed.1004463.s013.docx]

S1 Fig. Food purchases data linked to nutrition data from INTA (Institute of Nutrition and Food Technology) and Mintel by year, according to Chilean policy period.


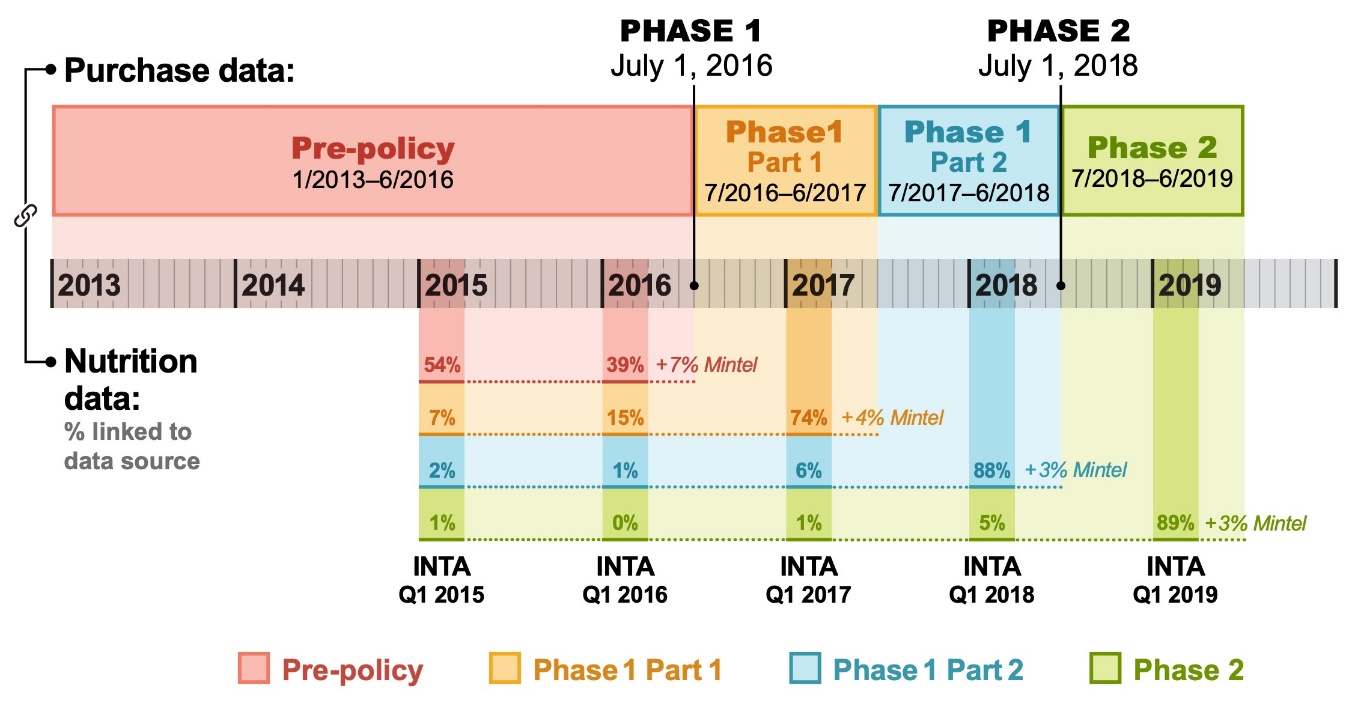


Note: Rows for nutrition data sum to 100% across rows (representing all nutrition data sources within a given policy period in the purchases data.
