# Supplementary material for: Decreases in purchases of energy, sodium, sugar, and saturated fat 3 years after implementation of the Chilean food labeling and marketing law: An interrupted time series analysis
Source: PLoS Med. 2024 Sep 27;21(9):e1004463. doi: 10.1371/journal.pmed.1004463 (PMC11432892; doi:10.1371/journal.pmed.1004463)
Supplement: S1 Appendix — (DOCX) [file pmed.1004463.s014.docx]

**S1 Appendix. Models and effects.**

This document presents the models and effects of interest. All outcomes were modeled using these specifications, except in the moderation analyses, where interaction terms were added between socioeconomic status and the trend and impact model parameters.

**Main model**

The conditional mean $\mu_{it}$ was modeled as:

$$\begin{aligned} \mu_{it}=g\left( \beta_{0}+\beta_{1}*t+\beta_{2}*post1_{t}+\beta_{3}*t*post1_{t}+\beta_{4}*post2_{t}+\beta_{5}*t*post2_{t}+\boldsymbol{X}_{it}\boldsymbol{\gamma} \right)\#\left( 1 \right) \end{aligned}$$

where $g$ is the exponential function, $post1_{t}$ equals 1 for $July 2016\leq t\leq June 2018$ and 0 otherwise, $post2_{t}$ equals 1 for $July 2018\leq t\leq June 2019$ and 0 otherwise, and $\boldsymbol{X}_{it}$ is a vector of control variables (see the text for details). In what follows, the $t$ subscript on $post1$ and $post2$ is omitted for brevity.

This model is a re-parameterization of the ‘canonical’ impact model with immediate intercept and slope changes:

$$\mu_{it}=g\left( \alpha_{0}+\alpha_{1}*t+\alpha_{2}*post1+\alpha_{3}*\left( t-t_{1} \right)*post1+\alpha_{4}*post2+\alpha_{5}*\left( t-t_{2} \right)*post2+\boldsymbol{X}_{it}\boldsymbol{\gamma} \right)$$

where $t_{1}=July 2016$ and $t_{2}=July 2018$, with:

$$\alpha_{0}=\beta_{0}$$

$$\alpha_{1}=\beta_{1}$$

$$\alpha_{2}=\beta_{2}+\beta_{3}*t_{1}$$

$$\alpha_{3}=\beta_{3}$$

$$\alpha_{4}=\beta_{4}+\beta_{5}*t_{2}$$

$$\alpha_{5}=\beta_{5}$$

The conditional means under no-policy ($post1=post2=0$), phase 1 ($post1=1$, $post2=0$), and phase 2 ($post1=0$, $post2=1$) conditions are given by, respectively:

$$\mu_{it}^{0}=\exp\left( \beta_{0}+\beta_{1}*t+\boldsymbol{X}_{it}\boldsymbol{\gamma} \right)$$

$$\mu_{it}^{1}=\exp\left( \beta_{0}+\beta_{2}+\left( \beta_{1}+\beta_{3} \right)*t+\boldsymbol{X}_{it}\boldsymbol{\gamma} \right)=\mu_{it}^{0}*\exp\left( \beta_{2}+\beta_{3}*t \right)$$

$$\mu_{it}^{2}=\exp\left( \beta_{0}+\beta_{4}+\left( \beta_{1}+\beta_{5} \right)*t+\boldsymbol{X}_{it}\boldsymbol{\gamma} \right)=\mu_{it}^{0}*\exp\left( \beta_{4}+\beta_{5}*t \right)$$

Hence the relative and absolute effects:

$$\frac{\mu_{it}^{1}}{\mu_{it}^{0}}=\exp\left( \beta_{2}+\beta_{3}*t \right)$$

$$\frac{\mu_{it}^{2}}{\mu_{it}^{0}}=\exp\left( \beta_{4}+\beta_{5}*t \right)$$

$$\mu_{it}^{1}-\mu_{it}^{0}=\mu_{it}^{1}-\frac{\mu_{it}^{1}}{\exp\left( \beta_{2}+\beta_{3}*t \right)}$$

$$\mu_{it}^{2}-\mu_{it}^{0}=\mu_{it}^{2}-\frac{\mu_{it}^{2}}{\exp\left( \beta_{4}+\beta_{5}*t \right)}$$

Thus, the relative effects depend on $t$ and the absolute effects depend on $i$ and $t$. These effects were summarized by taking the average relative effect over the phase 1 and phase 2 *months* (averaging over $t$), and the average absolute effects over the phase 1 and phase 2 subsamples (averaging over $i$ and $t$). For example, the average relative effect during phase 1 is:

$$\frac{1}{t_{2}-t_{1}}\sum_{t=t_{1}}^{t_{2}-1} \exp\left( \beta_{2}+\beta_{3}*t \right)$$

with the average relative effect during phase 2 obtained by replacing $t_{1}$ by $t_{2}$ and $t_{2}-1$ by $T$, the last period (and, equivalently, $t_{2}$ by $T+1$).

This model assumes:

1. The impact of the law was *proportional*, i.e. households which would have had different levels of the outcome in the event of no policy experienced different changes in absolute terms but equal changes in relative terms.
2. Each phase had an immediate effect, and that effect potentially increased or decreased *steadily* over time.

Assumption 1 is in contrast to the assumption of different changes in relative terms but equal changes in absolute terms, implied by the identity link function $g\left( z \right)=z$.

Assumption 2 defines the impact model. This particular impact model allows for time-invariant effects as a special case, but imposes the restriction that if the effects increased or decreased over time, they did so at a steady rate until the next phase started.

This model was estimated using the correlated random-effects (CRE) Poisson estimator with standard errors clustered at the household level to account for arbitrary intra-household correlation. The average effects and their standard errors were calculated as non-linear combinations of the parameters using Stata’s nlcom command (average relative effects) and as simple differences in the fitted values’ sample averages under pre-policy and phase 1 or phase 2 conditions estimated by Stata’s margins command (average absolute effects).

**Sensitivity analyses**

*Label rollout period*

Allowing for a label rollout period in the form of a temporary slope change in the trend between April and June 2016 yields the following modification to the main model:

$$\mu_{it}=g\left( \beta_{0}+\beta_{1}*t+\delta*t*rollout_{t}+\beta_{2}*post1_{t}+\beta_{3}*t*post1_{t}+\beta_{4}*post2_{t}+\beta_{5}*t*post2_{t}+\boldsymbol{X}_{it}\boldsymbol{\gamma} \right)$$

where $rollout_{t}$ equals 1 for $April 2016\leq t\leq June 2016$ *provided the policy went into effect* and 0 otherwise. In this model, the labels are progressively introduced to consumers as the non-labeled versions of high-in products are sold out or removed and replaced with their labeled versions, which we parameterize as a slope change in the trend without an intercept change.

Because $rollout_{t}$ equals 0 under both no-policy conditions and phase 1 and phase 2 conditions past the label rollout period, the conditional means and relative and absolute effects are unchanged.

*Quadratic trend*

The main model and its modification to allow for a label rollout period are easily extended to the case of a quadratic trend.

*Two-part model*

For the two-part model, the conditional mean was modeled as:

$$\begin{aligned} \mu_{it}=\Pr\left( y_{it}>0|t,post1, post2,\boldsymbol{X}_{it} \right)*\mu_{it}^{+} \end{aligned}$$

where $\mu_{it}^{+}$ is the conditional mean of $y_{it}>0$ and is given by (1).

The probability of a positive outcome was modeled as:

$$\Pr\left( y_{it}>0|t,post1, post2,\boldsymbol{X}_{it} \right)=\Lambda\left( \beta_{0}+\beta_{1}*t+\beta_{2}*post1+\beta_{3}*t*post1+\beta_{4}*post2+\beta_{5}*t*post2+\boldsymbol{X}_{it}\boldsymbol{\gamma} \right)$$

where $\Lambda$ is the cumulative logistic distribution:

$$\Lambda\left( z \right)=\frac{1}{1+\exp\left( -z \right)}$$

Therefore, the conditional mean is given by:

$$\mu_{it}=\frac{\exp\left( \beta_{0}+\beta_{1}*t+\beta_{2}*post1+\beta_{3}*t*post1+\beta_{4}*post2+\beta_{5}*t*post2+\boldsymbol{X}_{it}\boldsymbol{\gamma} \right)}{1+\exp\left( -\beta_{0}-\beta_{1}*t-\beta_{2}*post1-\beta_{3}*t*post1-\beta_{4}*post2-\beta_{5}*t*post2-\boldsymbol{X}_{it}\boldsymbol{\gamma} \right)}$$

The denominator of this expression leads to more complex relative and absolute effects than in the one-part model. In particular, the relative effects are no longer just a function of time. Therefore, we used Stata’s margins command to estimate the relative and absolute effects, averaged over the phase 1 and phase 2 subsamples.
