# Supplementary material for: Decreases in purchases of energy, sodium, sugar, and saturated fat 3 years after implementation of the Chilean food labeling and marketing law: An interrupted time series analysis
Source: PLoS Med. 2024 Sep 27;21(9):e1004463. doi: 10.1371/journal.pmed.1004463 (PMC11432892; doi:10.1371/journal.pmed.1004463)
Supplement: S2 Appendix — (DOCX) [file pmed.1004463.s015.docx]

**S2 Appendix. Differences between papers.**

| The overall pattern of results reported in the current study is consistent with our previously published evaluations of the first phase of the Chilean Law using Kantar WorldPanel data in that they show an overall decline in calories driven by a decline in High-in calories, with partial compensation by an increase in Not high-in calories. However, when comparing current Phase 1 results for total food and beverage purchases to our previously published results, there are several differences. Compared to our previously published results, declines in High-in calories purchased are smaller and increases in Not high-in calories purchased are also smaller, resulting in a larger estimation of declines in overall calories purchases. This pattern is consistent across nutrients, with the exception that for sugar, current results yield a larger decline in High-in calories from sugar than did previous results. There are multiple explanations for these findings relating to differences in the datasets used for these analyses. In our previous results, we applied Phase 1 cutoffs to classify High-in vs. Not high-in foods, whereas in our current results, we applied Phase 3 (more stringent) cut-offs across all phases in order to have a consistent definition of regulation status over time; these differences in classifications resulted in a higher proportion of foods being classified as “High-in” and less as “not high in” during Phase 1 in our current analysis. In our previous results, we used a shorter pre-policy window (January 1, 2015- June 2016) whereas in our current results, we used a longer pre-policy window (July 1, 2013-June 2016) in order to achieve balance with the longer post-policy window, since we were examining both Phase 1 and Phase 2. As shown in the sensitivity results, altering the pre-policy window changes the counterfactual, which is based on pre-existing trends, and can lead to estimation of larger or smaller differences between the observed and counterfactual. Similarly, our current results included the entire Phase 1 (July 1, 2016-June 30, 2018), and included updated Nutrition Facts Panel information collected in the first semester of 2018, whereas our previous results were truncated at December 31, 2017 due to data availability at the time of publication. It is possible that products were additionally reformulated in 2018 in anticipation of Phase 2 of the law, which was implemented in July 2018 and included stricter nutrient thresholds, which would likely lead to larger reductions in nutrients purchased. Finally, there were other differences between the analyses, including covariates (e.g., public holidays by region and month, inclusion of the SES variable), weighting technique (matching by production period vs. month), and model specifications, which reflect our updated understanding of the dataset. The differences in results highlights the need for careful attention to methodological issues such as how products are reviewed and linked to NFP data as well as how pre- and post-policy time windows are created. As policy implementation continues across the globe, it will be important to understand not only how policy-linked purchasing patterns change over time, but how to develop and implement methodological strategies for natural experiments to evaluate these. |
| --- |
